# Optogenetic delivery of trophic signals in a genetic model of Parkinson’s disease

**DOI:** 10.1371/journal.pgen.1009479

**Published:** 2021-04-15

**Authors:** Alvaro Ingles-Prieto, Nikolas Furthmann, Samuel H. Crossman, Alexandra-Madelaine Tichy, Nina Hoyer, Meike Petersen, Vanessa Zheden, Julia Biebl, Eva Reichhart, Attila Gyoergy, Daria E. Siekhaus, Peter Soba, Konstanze F. Winklhofer, Harald Janovjak

**Affiliations:** 1 Institute of Science and Technology Austria (IST Austria), Klosterneuburg, Austria; 2 Molecular Cell Biology, Institute of Biochemistry and Pathobiochemistry, Ruhr University Bochum, Bochum, Germany; 3 Australian Regenerative Medicine Institute (ARMI), Faculty of Medicine, Nursing and Health Sciences, Monash University, Clayton/Melbourne, Australia; 4 European Molecular Biology Laboratory Australia (EMBL Australia), Monash University, Clayton/Melbourne, Australia; 5 Center for Molecular Neurobiology (ZMNH), University Medical Center Hamburg-Eppendorf, Hamburg, Germany; Stanford University School of Medicine, UNITED STATES

## Abstract

Optogenetics has been harnessed to shed new mechanistic light on current and future therapeutic strategies. This has been to date achieved by the regulation of ion flow and electrical signals in neuronal cells and neural circuits that are known to be affected by disease. In contrast, the optogenetic delivery of trophic biochemical signals, which support cell survival and are implicated in degenerative disorders, has never been demonstrated in an animal model of disease. Here, we reengineered the human and *Drosophila melanogaster* REarranged during Transfection (hRET and dRET) receptors to be activated by light, creating one-component optogenetic tools termed Opto-hRET and Opto-dRET. Upon blue light stimulation, these receptors robustly induced the MAPK/ERK proliferative signaling pathway in cultured cells. In PINK1^B9^ flies that exhibit loss of PTEN-induced putative kinase 1 (*PINK1*), a kinase associated with familial Parkinson’s disease (PD), light activation of Opto-dRET suppressed mitochondrial defects, tissue degeneration and behavioral deficits. In human cells with PINK1 loss-of-function, mitochondrial fragmentation was rescued using Opto-dRET *via* the PI3K/NF-кB pathway. Our results demonstrate that a light-activated receptor can ameliorate disease hallmarks in a genetic model of PD. The optogenetic delivery of trophic signals is cell type-specific and reversible and thus has the potential to inspire novel strategies towards a spatio-temporal regulation of tissue repair.

## Author summary

The death of physiologically important cells and tissues underlies of a wide range of diseases, including the neurodegenerative disorder Parkinson’s disease. Currently, the two major strategies to counter cell degeneration are the injection of soluble growth factor peptides and growth factor gene therapy. Importantly, both strategies can lead to the undesired activation of healthy bystander cells or the non-natural permanent modification of cells and their internal signals. Here, we developed a light-based method to overcome these limitations. The use of optogenetics allowed delivering cell type-specific pro-survival signals in a genetic model of Parkinson’s disease. In *Drosophila* and human cells that exhibit loss of the PINK1 gene, akin to autosomal recessive Parkinson’s disease, we efficiently suppressed disease phenotypes using a light-activated tyrosine kinase receptor. This work demonstrates a ‘remote controlled’ and thus spatio-temporally precise strategy to interfere with degeneration and may open new avenues towards tissue repair in a variety of disease models, including but not limited to diseases of the brain.

## Introduction

Biology occurs over a wide range of time and length scales, from milliseconds and nanometers for protein folding, to days and centimeters for organism development. In recent years, powerful research methods have been developed that permit the manipulation of biological processes on even the smallest length and shortest time scales. In optogenetics, natural or reengineered photoreceptors are expressed in genetically defined cell populations to optically activate or inhibit, e.g., neuronal action potential firing or cell signaling. The use of light provides unprecedented precision in space and time as a way to answer previously unresolvable questions in a multitude of disciplines, including microbiology, cell/developmental biology, synthetic biology and neuroscience. In particular, spatio-temporally precise perturbation of selected cells in intact organisms can reveal cause-consequence-relationships that are a critical determination for understanding central nervous system function or animal development [1–3]. Optogenetics also provides access to the reversible and rapid activation of cell signaling pathways that is required for dissection of their dynamic properties [4,5] and for development of new drug discovery platforms [6]. Inspired by these successes, optogenetics is continuously translated into new research areas, including disease mechanism and therapy.

Shortly after its inception, optogenetics was beginning to be employed in the study of neural circuits that are known to be affected by neurological and neurodegenerative disorders, including spinal cord injury, stroke and Parkinson’s disease (PD) [7,8]. In this field, optogenetics has been mainly applied to shed new light on the mechanisms of currently utilized therapies (e.g., deep brain stimulation in PD) or of therapies of the future (e.g., stem cell-based tissue regeneration) [9,10]. This work was followed by the development of light-gated prosthetic approaches in which a genetically introduced photoreceptor senses either natural light, e.g. for optogenetic vision restoration [11] that has matured into human clinical trials [12], or light from a prosthetic source, e.g. for heart or skeletal muscle pacing in animal models [13,14]. Notably, these pioneering studies harnessed optogenetics to excite or inhibit electrical signals through regulated ion flow ions across the cell membrane. In apparent contrast, the optogenetic delivery of trophic signals, which support cell survival and are critical in the context of degeneration, has never been demonstrated in a disease model. It is unclear if this is feasible as hypo- or hyperactivity of pro-survival pathways is linked to undesired cellular outcomes (see below).

**Fig 1:**
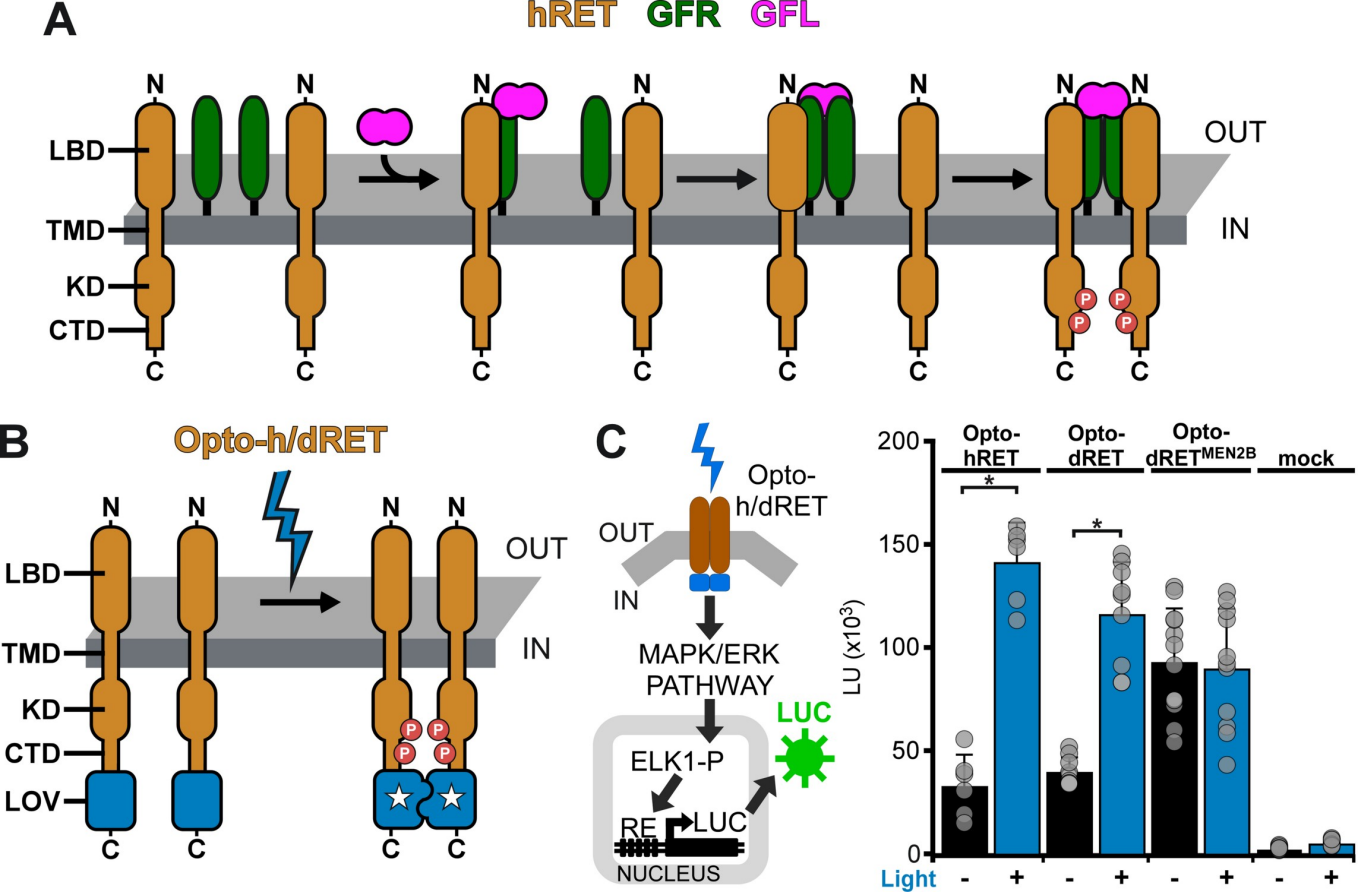
Engineering of light-activated RET receptors. (**A**) hRET and dRET consist of an extracellular ligand-binding domain (LBD), single-span transmembrane domain (TMD) and intracellular domain (KD: kinase domain, CTD: C-terminal tail domain). Activation by a GFL and GFRα was shown to result in the formation of a human ternary complex (binding model of Schlee *et al*. [40]). (**B**) In light-activated Opto-h/dRET, the LOV domain of the AUREOCHROME1 photoreceptor of *V*. *frigida* is incorporated at the receptor C-terminus. (**C**) MAPK/ERK pathway activation in response to blue light (I = 250 μW/cm^2^, λ = 470 nm, 8h continuous) for HEK293 cells transfected with *Opto-hRET*, *Opto-dRET* or *Opto-dRET*^*MEN2B*^. The MAPK/ERK reporter utilizes a pathway-specific Elk1 *trans*-activator to induce transcription of luciferase (LUC; see Materials and Methods for details). Light units (LU; mean ± SD) for dark treated cells and illuminated cells are given (n = 6 to 12, three independent experiments; t-test, *: p < .0001).

We and others have recently engineered light-activated variants of key signaling proteins that now provide a basis for the optogenetic delivery of trophic signals to animal models of disease. Particular engineering success was reported for receptor tyrosine kinases (RTKs) [15–19]. RTKs are expressed in virtually all human cell types and respond to growth factors (GFs) with conformational changes and/or oligomerization state changes that result in receptor *trans*-phosphorylation. *Trans*-phosphorylation is then followed by recruitment of intracellular secondary messengers in, e.g., the mitogen-activated protein kinase/extracellular signal-regulated kinase (MAPK/ERK) or phosphatidylinositol-3 kinase/AKT (PI3K/AKT) signaling pathways. Because of their ability to activate these proliferative and pro-survival pathways, RTKs are prime targets in several neurodegenerative disorders. In the context of PD, the RTK RET [20] has been intensively investigated in both preclinical and clinical studies. hRET is activated by glial cell line-derived neurotrophic factor (GDNF) family ligands (GFLs; these are GDNF, neurturin, artemin and persephin) that bind GDNF family receptor α (GFRα) co-receptors (GFR α1–4) to recruit dimeric RET into a ternary complex (Fig 1A). GFLs are linked to the development and maintenance of dopaminergic (DA) midbrain neurons and have been pursued as disease-modifying agents in PD, either by local injection or by gene delivery using adeno-associated viruses [21,22]. Despite initial success in animal models, outcomes in clinical trials were limited [23,24], which was attributed to difficulties in GFL delivery, limited responsiveness of the targeted DA neurons and advanced PD in some of the recruited patients. In addition, there are concerns that the continuous delivery of GFLs can lead to counter-productive compensatory effects [25–28]. These observations and considerations have highlighted a need for methods that can control the GFL-RET-axis in a reversible and more precise manner [29,30].

Here, we explored optogenetics as a means for delivery of trophic signals in a genetic model of PD. We first reengineered full-length hRET and its *Drosophila* orthologue dRET [31–33] to be activated by light in optogenetic tools termed Opto-hRET and Opto-dRET. We then showed that temporally precise dRET activation *in vivo* can be used to induce degeneration in a tissue sensitive to ectopic RTK signaling. Optogenetic delivery of RET signals was then successfully applied in a genetic fly model of PD. Mutations in the *PINK1* gene are linked to autosomal recessive PD [34–36], and *Drosophila* has been shown to be a suitable model to study consequences of PINK1 loss-of-function [37–39]. We suppressed *Drosophila* phenotypes associated with PINK1 deficiency and identified the involved downstream signaling pathways in a human cellular model. This work demonstrates the use of optogenetics as a cell-type specific and remote controlled method to exert beneficial trophic effects in a genetic disease model.

## Results

### Light-activated hRET and dRET receptors

hRET assembles in dimers in the activated ternary GFL_2_-GFRα_2_-RET_2_ complex [40,41] (Fig 1A), and forced dimerization by mutations or synthetic binding domains has been shown to induce signaling of hRET [42,43] and dRET [44,45]. Based on these observations, we converted hRET and dRET into optogenetic tools by incorporating a light-activated dimerization switch. To achieve this switch, we utilized the light-oxygen-voltage-sensing (LOV) domain of the AUREOCHROME1 photoreceptor from the yellow-green algae *Vaucheria frigida* (AU1-LOV) [46] (Fig 1B). AU1-LOV is a member of the large LOV domain superfamily and responds to blue light with formation of a symmetric homodimer [47] (S1 Fig). AU1-LOV is smaller than other photoreceptors commonly used in optogenetics (145 aa in length; this corresponds to ~a third of the length of cryptochromes or phytochromes) [48,49] and relaxes slower than many other LOV domains from the light-activated ‘lit’ state (that is predominantly dimeric) to the dark-adapted state (that is predominantly monomeric; relaxation time constant ~600 s) [15,50]. We and others have shown that small size and slow cycling make AU1-LOV well suited for activation of membrane receptors by enabling dimer assembly for a sufficient duration [15,18,51]. We placed AU1-LOV at the far C-terminus of the RET receptors because fluorescent proteins were previously incorporated at this site without negative impact on receptor signaling or trafficking [52,53]. To functionally test the generated Opto-hRET and Opto-dRET, we took advantage of the fact that *Drosophila* RTKs can couple to the mammalian MAPK/ERK pathway *via* Ras [45]. We and others utilize human embryonic kidney 293 (HEK293) cells for testing new optogenetic methods because these cells do not exhibit native light-induced signaling events. Using transcriptional reporters [15,48], we found robust induction of MAPK/ERK signaling upon blue light stimulation of HEK293 cells transfected with Opto-hRET and Opto-dRET (intensity (I) = 250 μW/cm^2^, wavelength (λ) = 470 nm) (Fig 1C). Whilst Opto-hRET activated transcriptional responses more strongly than Opto-dRET, Opto-dRET activation levels were comparable to those reached by the Opto-dRET^MEN2B^ variant (Fig 1C). Opto-dRET^MEN2B^ contains a Met to Thr gain-of-function substitution in the kinase domain that was discovered in multiple endocrine neoplasia (MEN) Type 2B as causative for potent receptor hyperactivation in the absence of GFLs [44,54]. Incorporation of AU1-LOV does not further induce RET signaling in the absence of light (S2 Fig) with basal signaling in this assay attributable to receptor overexpression. Collectively, these results show that cell signaling activity can be induced by blue light through Opto-h/dRET receptors.

### Opto-dRET function *in vivo*

**Fig 2:**
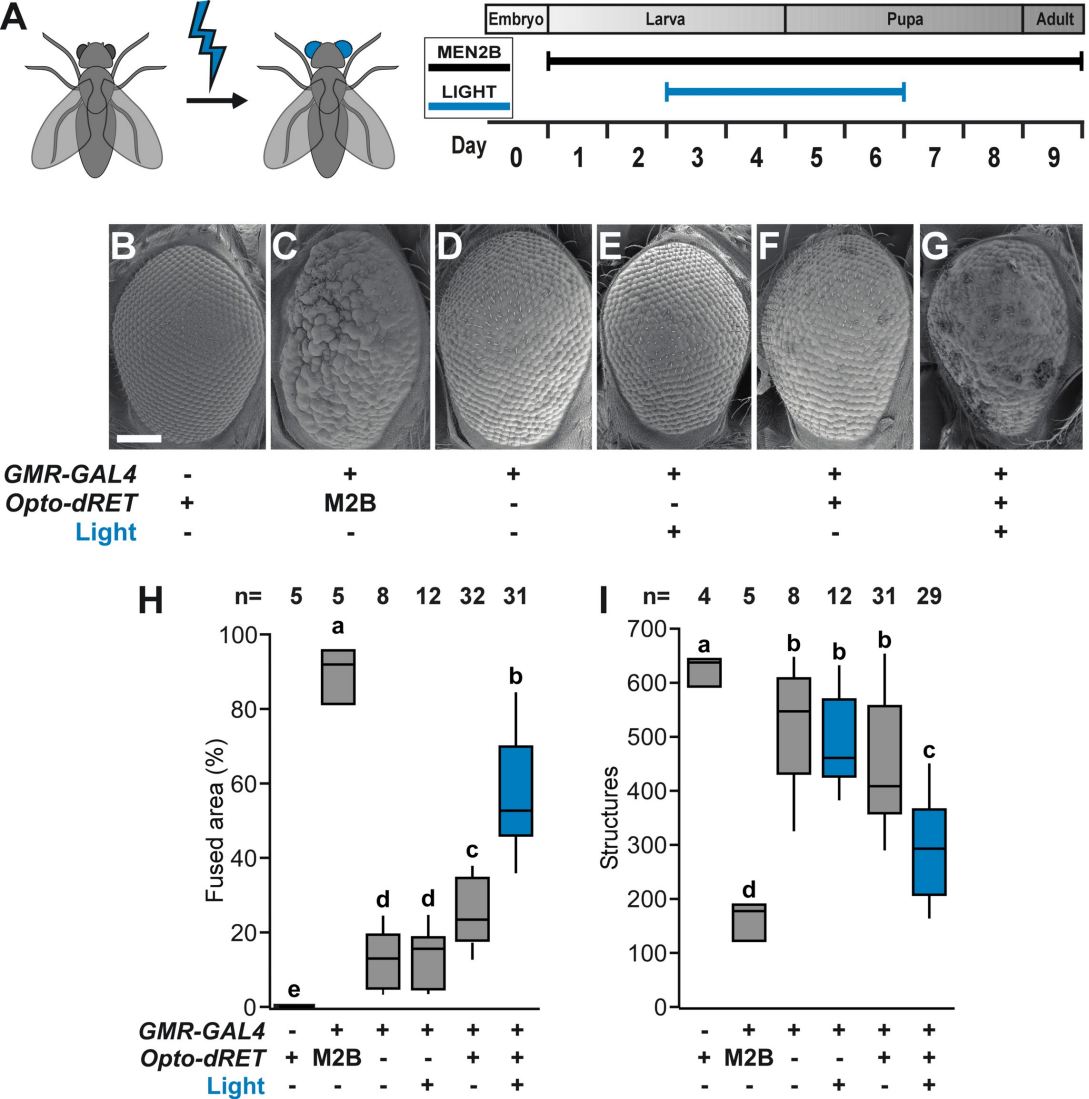
Induction of retina roughening and phenotype quantification. (**A**) Developmental time window targeted by light in retina experiments. (**B-G**) Representative retina SEM images. Scale bar: 0.1 mm. (**H** and **I**) Quantification of rough retina phenotypes of one-day old flies as fused area and the number of structures identified. “M2B” denotes Opto-dRET^MEN2B^. The number of analyzed flies is given (at least three independent experiments) and bars sharing the same label are not significantly different (ANOVA/Bonferroni corrected t-tests of means, p>.04). Continuous light intensity was 385 μW/cm^2^ for the duration shown in A.

We next tested if Opto-dRET can be applied *in vivo* to conduct a temporally-targeted gain-of-function experiment (Fig 2A). We choose the *Drosophila* retina for this experiment because RTKs and their downstream pathways are tightly regulated during its morphogenesis, and because RTK hyperactivation during retina development results in marked phenotypes. For instance, two RTKs, the *Drosophila* epidermal growth factor receptor (DER) and Sevenless, orchestrate retinal cell growth, differentiation and regulated death [55,56]. These RTKs act in late larval and early pupal stages to form the fourteen cells that compose each ommatidium unit eye and the ommatidial lattice [57]. Hyperactivation of RTK signaling during these stages has been shown to result in irregular ommatidia size and spacing leading to a disrupted tissue pattern termed ‘roughening’ [58]. We generated transgenic flies in which the *Opto-dRET* gene is placed downstream of five *UAS* elements (S3 Fig). We also generated analogous flies expressing the constitutively-active Opto-dRET^MEN2B^. We then targeted Opto-dRET or Opto-dRET^MEN2B^ to the retina using the *GMR-GAL4* driver, which induces expression in cells located posterior of a morphogenetic furrow that sweeps in anterior direction to initiate mitosis and cell differentiation [56]. In Opto-dRET^MEN2B^ flies, scanning electron microscopy (SEM) revealed a marked rough retina phenotype (compare Fig 2B and 2C). Roughening was previously observed in flies expressing dRET^MEN2B^ [44], and based on the severe outcome observed for Opto-dRET^MEN2B^ we concluded that AU1-LOV attachment does not negatively impact receptor function. We next illuminated control flies and Opto-dRET flies in a time window that captures ommatidia and lattice formation (from third instar larva through to the second day after pupariation; I = 385 μW/cm^2^, λ = 470 nm; Fig 2A). In control flies without Opto-dRET, we did not observe light-induced roughening indicating that light alone did not have an effect on the retina (compare Fig 2D and 2E; in agreement with previous studies, we observed mild phenotypes upon GAL4 expression with the *GMR* driver [59]). In apparent contrast, we found that light stimulation resulted in a marked roughening in Opto-dRET flies (compare Fig 2F and 2G). To quantify this effect, we manually metered in each retina image the ‘fused area’ (the area without identifiable ommatidia) and also applied computational methods to count individual ommatidia (~600 ommatidia can be assigned in our frontal view images) as two measures of tissue integrity. We found that upon illumination the fused area increased and the number of identified structures decreased specifically in the illuminated Opto-dRET flies (Fig 2H and 2I). For these and control flies, we also determined lattice regularity, which is defined as the ratio of the mean and the standard deviation (SD) of the ommatidia nearest-neighbor distance (NND) distribution [60]. Regularity decreased from 3.98 ± 0.39 in WT flies to 2.15 ± 0.55 in illuminated Opto-dRET flies, and these values are indicative of near-perfect regularity and near-random assembly, respectively [61]. In a complementary experiment, we illuminated flies onwards of the second day after pupariation, which we expected to result in a reduced phenotype as this time window follows formation of the basic lattice [57]. Indeed, we did not observe large areas of disorganized tissue but rather a similar number of structures as in the unilluminated flies (S4 Fig), demonstrating that timed light stimulation in the earlier time window allowed targeting tissue stages that possess high sensitivity to ectopic signals [62,63]. The potent effects induced by Opto-dRET upon light stimulation establishes the suitability of this approach to modify tissue structure and development *in vivo*.

### Suppression of defects in a genetic model of PD

**Fig 3:**
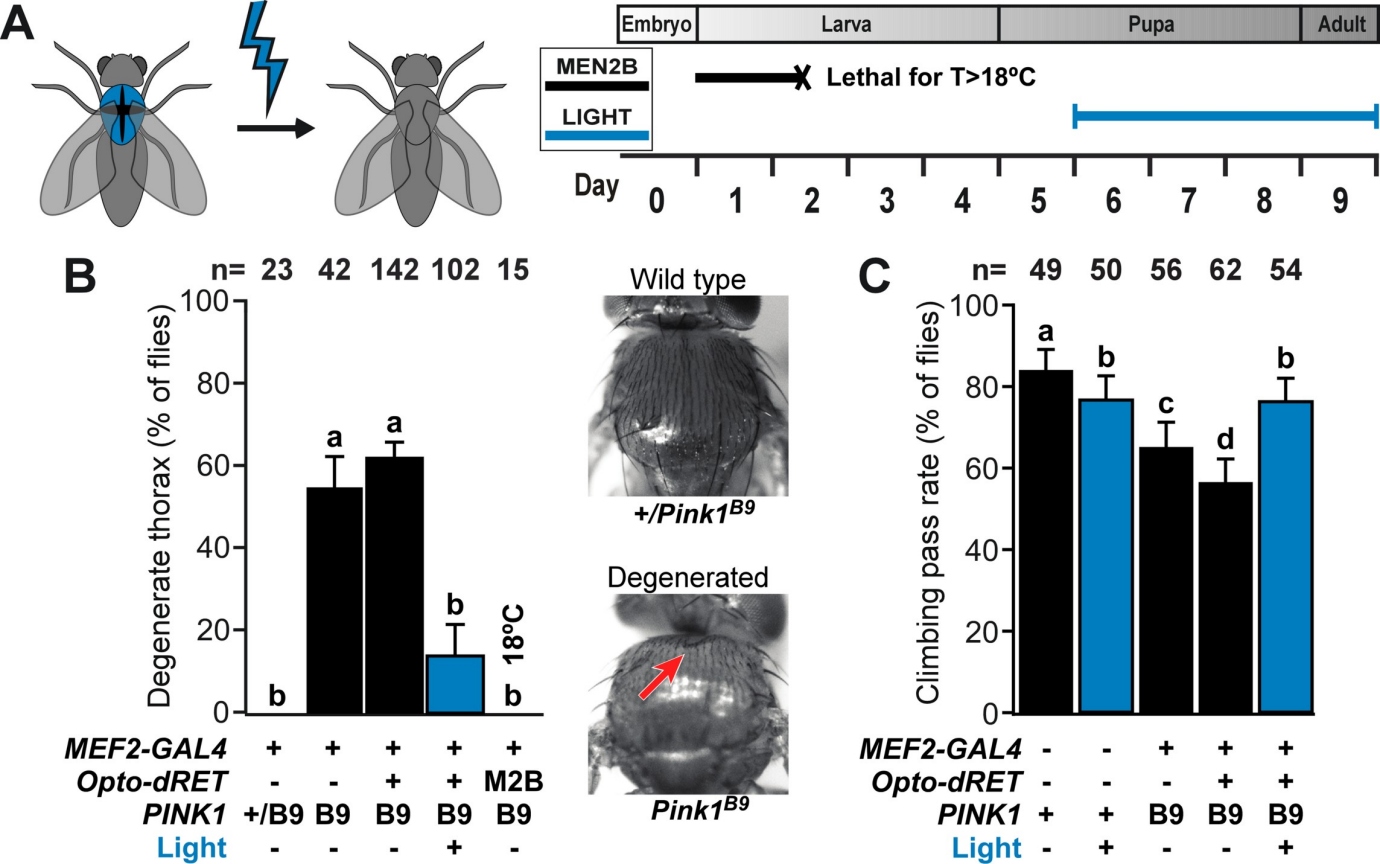
Suppression of thorax defects and locomotion deficits. (**A**) Time window targeted by light in experiments with PINK1^B9^ flies. Illumination of pupal and adult stages prevented lethality observed upon Opto-dRET signaling in earlier stages (e.g., Opto-dRET^MEN2B^ flies were grown at 18°C to prevent lethality during development; see Main Text). (**B**) Percentage of flies with a degenerate thorax phenotype. Representative bright field thorax images shown on the right. Hollow thorax is highlighted by the red arrow. (**C**) Climbing ability of flies. “M2B” denotes Opto-dRET^MEN2B^. *PINK1* “+” denotes the WT gene. For B and C, counts ± SE for the indicated number of flies (n) is given. Percentages sharing the same label are not significantly different (Fisher’s exact test, p>.04). Continuous light intensity was 320 μW/cm^2^ for the duration shown in A.

With Opto-dRET in hand, we went on to explore if defects in a genetic disease model can be ameliorated using optogenetics (Fig 3A). PINK1 is a Ser/Thr kinase that localizes to mitochondria and supports their integrity and function. Loss-of-function mutations and dominant negative mutations in the *PINK1* gene are associated with autosomal recessive PD [34–36]. In *Drosophila*, loss of X-linked *PINK1* leads to a striking phenotype, including tissue degeneration, locomotor defects and disruption of mitochondrial structure and function [64–67]. To test if optogenetics can suppress phenotypes associated with PINK1 deficiency, we expressed Opto-dRET in indirect flight muscles (IFMs) of PINK1^B9^ flies [64] using the *MEF2-GAL4* driver [68]. IFMs are frequently studied in *Drosophila* models of PD, and PINK1 loss-of-function leads to a marked ‘crushed’ thorax phenotype and reduced locomotion. We first compared PINK1^B9^ flies to Opto-dRET PINK1^B9^ flies that were not illuminated. Similar penetrance of thoracic defects (58 and 61% of flies exhibited a crushed thorax, respectively) showed that the engineered optogenetic receptor did not affect the phenotype in the absence of light (Fig 3B). When proceeding to light stimulation, we took into consideration that the opaque case and cuticle of pupa and adults may reduce blue light penetration to IFMs. To address this, we first confirmed that AU1-LOV can be activated by light of 1–5 μW/cm^2^ intensity, which corresponds to the product of minimal blue light transmission through the case or cuticle (~0.5% [69,70]) and the light intensity applied in our light chambers (I = 320 μW/cm^2^; S5A Fig). We observed that light of this intensity is indeed sufficient to activate AU1-LOV (S5B Fig). We then went on to light stimulate Opto-dRET PINK1^B9^ flies during late pupal stages and adult states (Fig 3A) (these stages coincide with the onset of degeneration [67,71]). Strikingly, we observed phenotype suppression in Opto-dRET PINK1^B9^ flies resulting in only 16% of flies with thoracic defects (Fig 3B; light stimulation in the absence of Opto-dRET did not alter phenotypes, S6 Fig). This result indicates marked improvement in tissue integrity upon light activation of Opto-dRET that was comparable to the improvement observed previously upon PINK1 overexpression in the PINK1^B9^ model [64]. We also tested if illumination restored the climbing deficits that accompany PINK1 loss-of-function. This was indeed the case with illuminated Opto-dRET flies but not illuminated control flies reaching climbing pass rates similar to those of WT flies (Figs 3C and S7).

**Fig 4:**
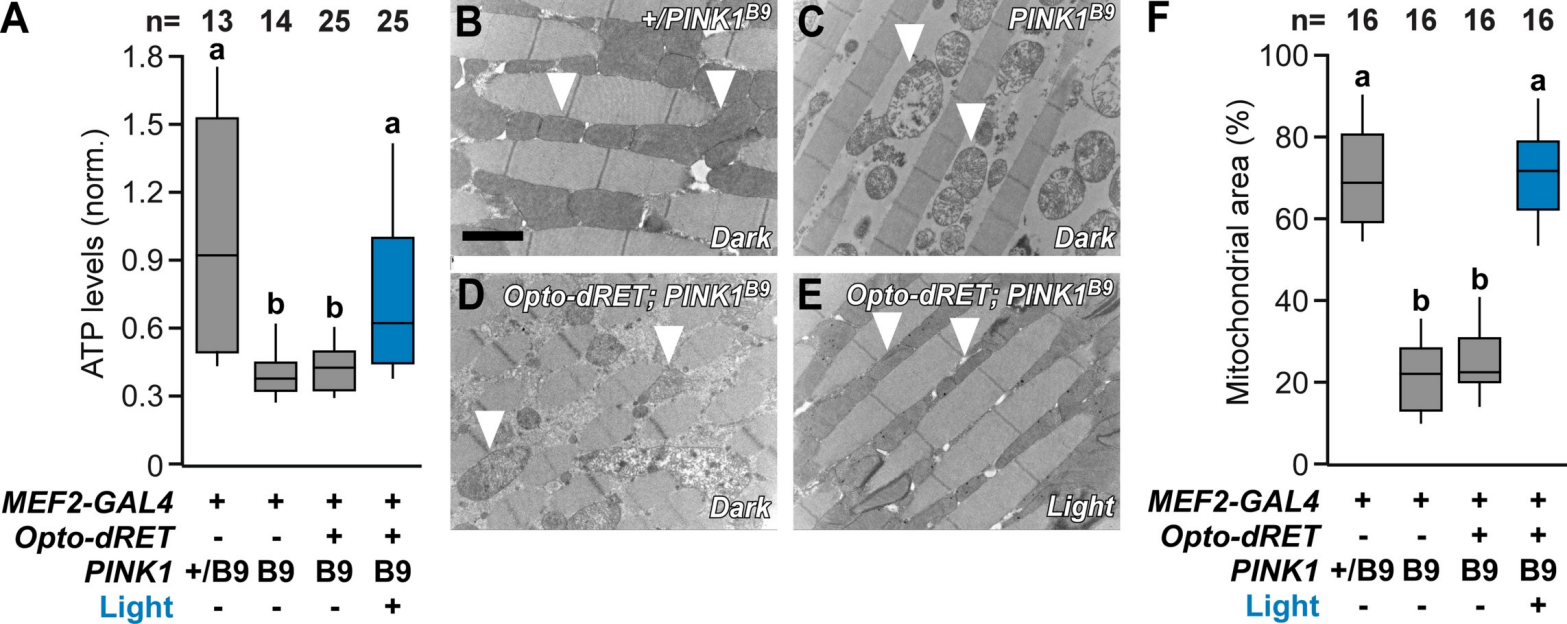
Improved mitochondrial structure and function. (**A**) ATP content in fly thoraces from PINK1^B9^ flies at the indicated conditions (normalized to the mean for control flies shown as bar 1). (**B-E**) Representative TEM images of thoracic indirect flight muscles. Arrow heads indicate mitochondria that are either electron dense (B: controls, E: illuminated PINK1^B9^ Opto-dRET flies) or malformed with disintegrated cristae (C: PINK1^B9^ flies, D: PINK1^B9^ Opto-dRET flies in the absence of light). Scale bar: 2 μm. (**F**) Analysis of mitochondrial density in TEM images. “M2B” denotes Opto-dRET^MEN2B^. *PINK1* “+” denotes the WT gene. In A, the number of analyzed flies is given (at least three independent experiments) and bars sharing the same label are not significantly different (ANOVA/Bonferroni corrected t-tests of means, p>.04). In F, the number of analyzed micrographs is given (at least three independent experiments) and bars sharing the same label are not significantly different (ANOVA/Bonferroni corrected t-tests of means, p>.04). Continuous light intensity was 320 μW/cm^2^ for the duration shown in Fig 3A.

Mitochondrial dysfunction is a major pathological alteration observed in sporadic and familial PD and also the primary cellular consequence of loss of PINK1. We therefore tested the effect of illumination on mitochondrial function and integrity in Opto-dRET PINK1^B9^ flies. PINK1^B9^ flies exhibited reduced muscle ATP levels and these levels could be restored by Opto-dRET and light stimulation (Figs 4A and S8). To examine mitochondrial integrity, we conducted ultrastructure analysis using transmission electron microscopy (TEM). PINK1^B9^ muscles exhibited broadening of the myofibril Z-line and enlarged mitochondria with fragmented cristae (compare Fig 4B and 4C). Illumination of Opto-dRET PINK1^B9^ flies was clearly beneficial with a reduced fraction of impaired mitochondria and an increased fraction of mitochondria with WT-like cristae structure (compare Fig 4D and 4E) that approached levels of control flies (Fig 4F). Overall, these results on the cell- and tissue-level demonstrate optogenetic suppression of consequences of PINK1 loss-of-function in a *Drosophila* model. We took advantage of temporally precise light stimulation to prevent undesired effects of continuous growth signal delivery. In this model specifically, one such effect is lethality upon dRET overactivation in muscle at early developmental stages [72], an outcome that we were able to prevent.

### Amelioration of mitochondrial defects in PINK1-deficient human cells

**Fig 5:**
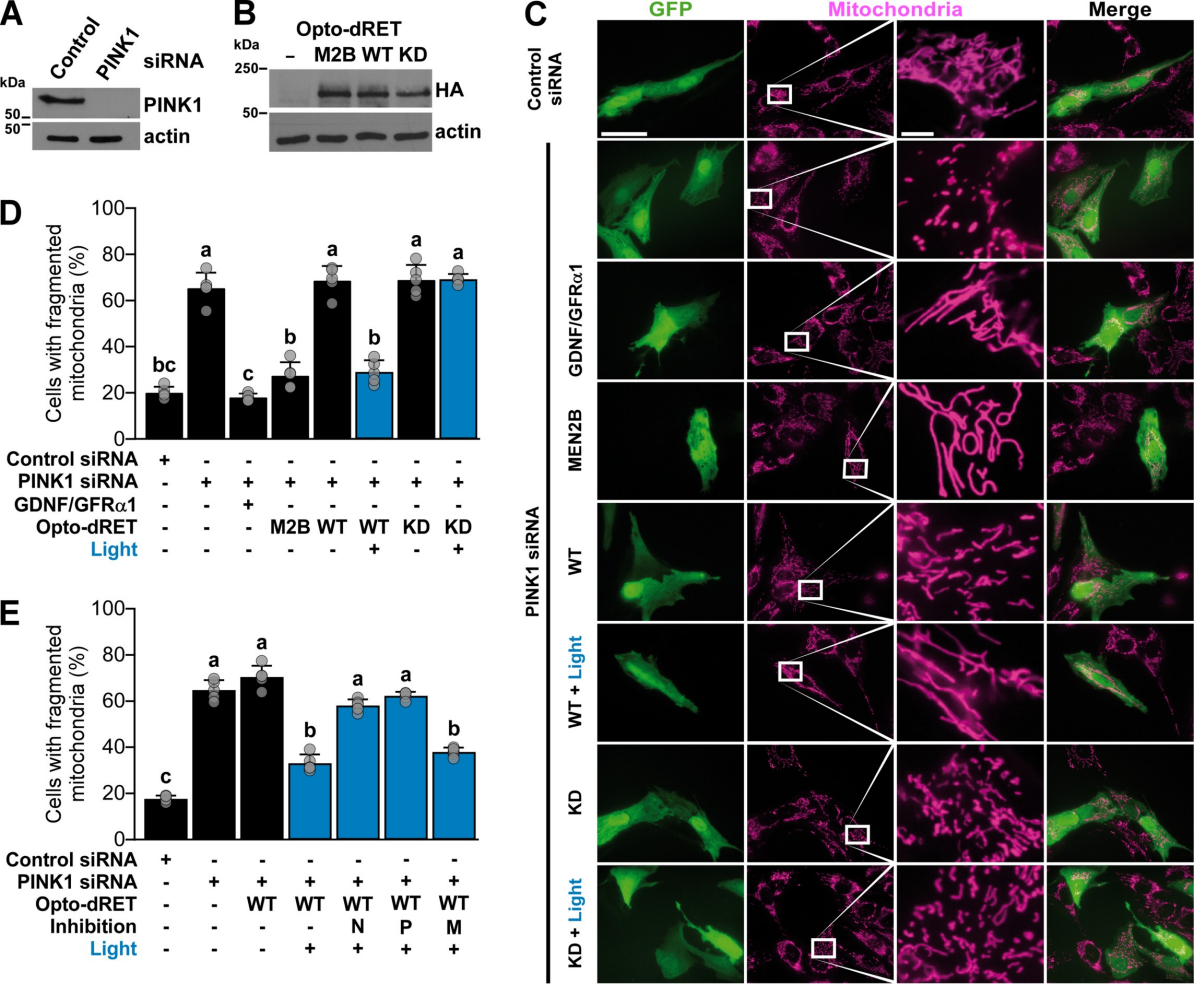
Rescue of mitochondrial fragmentation in human cells. (**A** and **B**) WB analysis of *PINK1* knock-down by siRNA and Opto-dRET expression. (**C**) Representative images for fragmentation of mitochondria induced by *PINK1* silencing. Magenta: MitoTracker. Green: GFP marker. Scale bar: 200 (columns 1, 2) or 20 μm (columns 3, 4). (**D**) Quantification of mitochondrial fragmentation upon light stimulation of RET, Opto-dRET, Opto-dRET^MEN2B^ or Opto-dRET^KD^ (I = 232 μW/cm^2^, λ = 470 nm, 4 h continuous). (**E**) Quantification analysis of mitochondrial fragmentation upon light activation of Opto-dRET and inhibition of NF-кB signaling (by IκB-2S/A, label “N”), PI3K (by LY294002, label “P”) or MEK1 (by PD98059, label “M”). For D and E, mean ± SD for five independent experiments is given (at least 150 cells per condition in each experiment). Means sharing the same label are not significantly different (ANOVA/Bonferroni corrected t-tests, p>.04).

Finally, we tested if light activation of RET signaling can revert defects induced by loss of PINK1 in human cells. We performed these experiments in dopaminergic neuroblastoma SH-SY5Y cells that have been previously applied to study how gene modifications observed in PD, including those in the *PINK1* gene [73], impact mitochondrial integrity. We transfected the cells with either control siRNA or *PINK1* siRNA as well as expression vectors for Opto-dRET, Opto-dRET^MEN2B^ or an inactive ‘kinase-dead’ (KD) Opto-dRET (Opto-dRET^KD^). Western blot (WB) analysis revealed efficient downregulation of PINK1 levels (Fig 5A) and that expression levels of the Opto-dRET variants were comparable (Fig 5B). Silencing of the *PINK1* gene resulted in severe mitochondrial defects with ~65% of cells exhibiting fragmented mitochondria (Fig 5C, rows 1 and 2, Fig 5D, bars 1 and 2). As shown previously, mitochondrial integrity was restored in this model through endogenous RET stimulated with GDNF/GFRα1 for 4 h [72,74]. In this paradigm, the fraction of cells with fragmented mitochondria was reduced to 20%, which is comparable to cells treated with control siRNA (Fig 5C, rows 1 and 3, Fig 5D, bars 1 and 3). We then analyzed Opto-dRET^MEN2B^ and Opto-dRET cells and found that either expression of Opto-dRET^MEN2B^ or light stimulation of Opto-dRET cells (I = 232 μW/cm^2^, λ = 470 nm, 4 h) rescued mitochondria with similar efficiency (~25% of cells displaying fragmentation; Fig 5C, rows 4 to 6, Fig 5D, bars 4 to 6). Similarly to the *Drosophila* experiments, no rescue was observed upon Opto-dRET expression in dark conditions, indicating limited basal activity in the absence of the light stimulus (Fig 5C, rows 2 and 5, Fig 5D, bars 2 and 5). We verified that the kinase activity of dRET is required for rescue (Fig 5C, rows 5 to 8, Fig 5D, bars 5 to 8) and that light alone did not influence mitochondrial morphology (S9 Fig). We also tested which signaling pathways downstream of RET are involved in mediating mitochondrial integrity. Of the main pathways activated by RET, we found that reversion of mitochondrial fragmentation depended on both the PI3K and nuclear factor ’kappa-light-chain-enhancer’ of activated B-cells (NF-кB) pathway, but not on the MAPK/ERK pathway (Fig 5E). This result is in agreement with previous studies showing that the protein network regulated by GFL/RET overlaps with that involved in PINK1 function [72,74]. Collectively, these findings demonstrate that beneficial trophic signals can be delivered to a human cellular model of PD using optogenetics.

## Discussion

Choreographed signaling of GFs and their cognate RTKs underlies tissue morphogenesis and homeostasis, whereas their aberrant activity is linked to human disorders. For instance, in the case of RET, gain-of-function is implicated in several forms of cancer and loss-of-function is linked to developmental disorders and neurodegeneration [75,76]. Motivated by the importance of RET, we engineered human and *Drosophila* RET receptors that can be activated by light. Recent work has demonstrated light activation of RTKs generally following seminal designs that built on either dimerizing [15] or oligomerizing [16] photoreceptor domains. We developed Opto-hRET and Opto-dRET using the homodimerizing AU1-LOV domain, and whilst LOV domains have enabled dimerization of isolated kinase domains in the past, we here show that this approach is suited for activation of full-length RET receptors. This suggests that enforced association at the RET C-terminus can overcome autoinhibition by elements of the extracellular domain that can counteract ligand-independent dimerization. These light-activated human and *Drosophila* RTKs add to an optogenetic arsenal that already consists of light-activated enzymes and optically-recruited signaling proteins, some of which have already permitted the optogenetic control of cell behavior in *Drosophila* [1,2].

In the first experiment, we applied Opto-dRET in the *Drosophila* retina to interfere with tissue morphogenesis. Retina development depends on concerted cell proliferation and differentiation events, and RTKs play a key role in ommatidia formation and ommatidial lattice generation. We observed retina malformations specifically in flies that were illuminated during tissue formation stages, in agreement with earlier observations that downstream pathways are not operating at maximal levels during retina development because of multiple and reiterative uses of RTKs [55,56,77]. This experiment complements recent optogenetic studies in *Drosophila* tissues other than the retina that have incorporated spatio-temporal regulation to identify tissues and stages with high sensitivity to ectopic signals [62,63,78,79].

We then explored if optogenetics can suppress phenotypes in a genetic animal model of PD. Previous optogenetic studies in the context of neurological and neurodegenerative disorders were mechanistic and focused on understanding or correcting aberrant electrical activity in excitable cells [7,8], whilst our goal was the delivery of trophic effects through the optical control of biochemical pro-survival pathways. Our model was *Drosophila* with loss of PINK1, a Ser/Thr kinase that causes autosomal recessive PD [34–36]. Although evidently not able to recapitulate all features of more complex animal models or the human disorder, we chose *Drosophila* as the model because PINK1 loss-of-function manifests in robust phenotypes that have previously helped delineate pathways implicated in mitochondrial physiology and in PD pathogenesis [64,67,71,80–87]. Cell degeneration in this model occurs most strongly in cells outside of the nervous system, such as in IFMs, likely because of their high energy demand. Activation of Opto-dRET resulted in efficient suppression of mitochondrial alterations, tissue degeneration and attendant locomotion fitness. We also demonstrated rescue of mitochondrial morphology in PINK1-deficient human cells. This second model allowed us to identify signaling pathways downstream of dRET that are essential for reversion of the defects, and PI3K and NF-кB activity were required to reestablish the healthy mitochondrial network. These pathways are known to act as an important node of crosstalk downstream of tyrosine kinases [88–90], and their involvement is in line with previous observations that the protein networks regulated by GFL/RET integrate with those altered in PD [72,74]. We noted that PINK1 deficiency phenotypes in flies and human cells were only modified by Opto-dRET upon stimulation with light but not in the dark, indicating little background activity of the receptor in the absence of activation of the introduced optical switch. It will be the next step to apply Opto-dRET in mammalian *in vivo* models of PD, which will require dosed gene delivery across targeted cell populations to avoid overexpression that may result in uncontrolled signaling pathway activation in this and also other optogenetic methods.

The new ability to remotely and spatio-temporally control cellular events relevant to human disease has previously inspired the pursuit of optogenetics-based treatment strategies, some of which have matured into clinical trials (see above). Translation of optogenetics into therapy is without question challenging, because of the need for light delivery into soft tissue and because of the non-human origins of many optogenetic proteins leading to potential immunogenicity. From a disease mechanism perspective, pro-survival signals, such as those elicited by GFLs, have been extensively studied in the context of degenerative disorders, and open mechanistic questions remain that may benefit from optogenetic technology in animal models. In PD, these questions include how the cellular signaling machinery of degenerating cells responds to GFLs, e.g. because of impaired RTK retrograde trafficking or expression, and how circuit function depends on the activation of different cell targets [29,30]. Cell type-specific optogenetic control may help to address these questions. Interestingly, it has been recently demonstrated that RET dysfunction in a mouse model of PD can be compensated by virally delivered RET [91]. This finding provides an encouraging basis for further exploration of Opto-RETs in mammalian models of PD. The properties of optogenetics mentioned above may also be more broadly harnessed to study tissue regeneration. For instance, many GF receptors have widespread tissue distribution and thus systemic growth factor administration may result in toxicity or undesired proliferation effects in cells other than those targeted [92]. Other GFs in turn exhibit limited half-life in the circulation or do not reach target tissues [93,94]. In this study, we demonstrated in a genetic model of PD that ligand-independent optical delivery of trophic signals is in principle possible, paving the way for future studies in animal models of PD and potentially also in models of other disorders linked to the GF-RTK axis.

## Materials and methods

### Engineering light-activated RET receptors

The gene encoding full-length dRET with the MEN2B M955T substitution (*dRET*^*MEN2B*^, a kind gift of Ross Cagan, Icahn School of Medicine at Mount Sinai, NY) was amplified from an expression vector by PCR and inserted into pUAST. To obtain *Opto-dRET*^*MEN2B*^ in pUAST, *AU1-LOV* [15] was inserted at the far C-terminus of the receptor. To obtain *Opto-dRET* in pUAST, the M955T substitution of *dRET*^*MEN2B*^ was reverted using site-directed mutagenesis. To express Opto-dRET in mammalian cells, the gene was amplified by PCR and sub-cloned into pcDNA3.1(-) including a hemagglutinin (HA)-epitope. To obtain *Opto-dRET*^*MEN2B*^ in pcDNA3.1(-), the M955T substitution was introduced using site-directed mutagenesis. To obtain the KD variant, the K805M substitution was introduced using site-directed mutagenesis. To express Opto-hRET and hRET-FKBP in mammalian cells, the full-length RET receptor was inserted into a pcDNA3.1(-) vector containing *AU1-LOV* or the engineered FKBP-F36V domain [15,95]. All constructs were verified by DNA sequencing. Sequences of the receptors are given in S1, S2 and S3 Tables.

### Cell culture, transfection and MAPK/ERK pathway activation (HEK293)

The MAPK/ERK pathway was assayed in HEK293 cells with the Elk1 *trans*-reporting system (PathDetect, Agilent). This system utilizes a unique fusion *trans*-activator consisting of Elk1 and the yeast GAL4 DNA binding domain, which upon phosphorylation by ERK induces transcription of the luciferase gene downstream of five UAS elements. The luciferase protein product is then detected as described below. HEK293 cells were maintained in DMEM supplemented with 10% FBS, 100 U/ml penicillin and 0.1 mg/ml streptomycin in a humidified incubator (37°C, 5% CO_2_). 50’000 cells were seeded in each well of white clear bottom 96-well plates (triplicates for each construct) coated with poly-*L*-ornithine (Sigma). Cells were reverse transfected with 3 to 25 ng receptor vector and ~200 ng combined reporting system vectors (fusion *trans*-activator vector and luciferase vector at a ratio of 1:10) per well using polyethylenimine (Polysciences). Six h after transfection, medium was replaced with CO_2_-independent or regular reduced serum starve medium (Gibco/Life Technologies; supplemented with 0.5% FBS, 2 mM L-Glutamine, 100 U/ml penicillin and 0.1 mg/ml streptomycin). Cells were then either illuminated with blue light in a custom incubator (PT2499, ExoTerra) or in a regular tissue culture incubator for 8 h or protected from light with foil as described previously [96]. FKBP-fusion receptors were activated with 10 nM AP20187 (Clontech). After incubation, plates were processed with a luciferase assay using standard reagents and luminescence was detected in a microplate reader (Synergy H1, BioTek or CLARIOstar, BMG Labtech). Low-light stimulation (S5B Fig) was performed as previously described [48] using a light blocking sample with an optical density of 1 and an external light intensity of 15 μW/cm^2^ (resulting in a final intensity of 1.5 μW/cm^2^).

### Cell culture, RNA interference, transfection and treatments (SH-SY5Y cells)

SH-SY5Y cells (DSMZ ACC 209) were maintained in DEMEM/F-12 (1:1) supplemented with 15% FBS (Sigma), 1% non-essential amino acid solution, 100 U/ml penicillin and 100 μg/ml streptomycin (Life Technologies) in a humidified incubator (37°C, 5% CO_2_). 1.5 x 10^5^ cells were seeded in each well of a 6-well plate containing two 15 mm coverslips per well. Transient co-transfection of siRNA oligos and DNA plasmids were performed using Lipofectamine 2000 (Thermo Fisher). The following three *PINK1* siRNAs were used at a final concentration of 60 pmol/ml each: siRNA *PINK1* HSS127945/127946/185707 (Life Technologies). To identify transfected cells by fluorescence microscopy, a plasmid encoding GFP was co-transfected (0.2 μg/well; in total, 1.2 μg/well were transfected). Mitochondrial morphology was analyzed 2 days after transfection as described below. For illumination of the cells, the 6-well plate was placed in a LED illumination unit inside the incubator. Cells were illuminated for 4 h at a wavelength of 470 nm and an intensity of 232 μW/cm^2^. To activate endogenous RET, cells were treated with recombinant human GDNF (Shenandoah Biotechnology) and human GFRα1 (R&D Systems) for 4 h at a final concentration of 100 ng/ml. Signaling pathway inhibitors were added to cells 1 h prior to illumination at the following concentrations: 50 μM LY294002 (PI3K inhibitor, Cell Signaling) or 30 μM PD98059 (MEK1 inhibitor, Cell Signaling). The HA-IκB-2S32A/S36A plasmid (IκB-2S/A [97]) was generated by overlap extension PCR using the following primers: mut-IκB-2S-fwd CCACGACGCCGGCCTGGACGCCATGAAAG, mut-IκB-2S-rev CGTCTTTCATGGCGTCCAGGCCGGCGTCG, BamHI-IκB2S-fwd ATATGGATCCTTCCAGGCGGCCGAGCGCCCCCAGGAG and IκB2S-NotI-rev ATATGCGGCCGCCTATAACGTCAGACGCTGGCCTCCAAACACACAGTC. The amplified fragments were digested with BamHI and NotI and cloned into the pcDNA3.1-N-HA vector. pEGFP-N3 was purchased from Clontech.

### Analysis of mitochondrial morphology (SH-SY5Y cells)

Mitochondria in SH-SY5Y cells growing on 15 mm coverslips were stained for 15 min with 25 nM MitoTracker red CMXRos (Life Technologies) diluted in cell culture media and then washed twice with medium. Mitochondrial morphology of living cells was immediately analyzed with a fluorescence microscope (Nikon Eclipse E400). Cells displaying an intact network of tubular mitochondria were classified as tubular. When this network was disrupted and mitochondria appeared either globular or rod-like, they were classified as fragmented [73]. For quantification of mitochondrial morphology, five independent experiments were performed. At least 150 transfected cells were analyzed per condition for each experiment.

### WB analysis (SH-SY5Y cells)

SH-SY5Y cells were analyzed two days after transient transfection for expression of Opto-dRET constructs and *PINK1* silencing efficiency. For stabilization of endogenous full-length PINK1, cells were treated with 10 μM FCCP (Agilent) for 2 h before cell lysis. Proteins were detected by WB using a monoclonal rabbit anti-PINK1 antibody (1:1000; Cell Signaling, D8G3) or an anti-HA antibody (1:1000; Covance, 16B12) for the Opto-dRET constructs. Data were normalized to monoclonal mouse anti-β-actin staining (1:2000; Sigma, AC-74).

### Fly strains, maintenance and scoring

Flies were raised on standard agar, cornmeal and molasses substrate supplemented with 1.5% nipagin. *GMR-GAL4* flies were a kind gift of Ross Cagan. *PINK1*^*B9*^*/FM6; MEF2-GAL4* flies were a gift of Alex Whitworth (University of Cambridge, UK). The PINK1^B9^ allele encompasses a 570 bp deletion from the second exon to the fourth exon and results in a complete loss of PINK1 protein [64]. Transgenic flies expressing Opto-dRET and Opto-dRET^MEN2B^ were generated by injection of pUAST receptor constructs (BestGene). For selection, balanced fly lines (~12 transformants/line) were crossed with *GMR-GAL4* flies. Approximately 10 days after crossing, descendants were visually inspected for the presence of a rough retina phenotype. Rough retina and hollow thorax phenotypes were scored in adult flies (at least two days old) on a dissecting microscope equipped with a digital camera (M205FA and DFC3000G, Leica Microsystems). Genotypes of fly lines utilized in this study are summarized in S4 Table.

### Light stimulation of flies

Flies were illuminated inside their vials in the custom LED incubator (S5A Fig) set to the temperature and light intensities indicated in the text and figures. Vials containing control flies were wrapped with foil and placed in the same incubator. Light incubators were placed in a controlled environment to maintain humidity at 65%. Experiments with GAL4 drivers were conducted at 29°C to increase receptor expression.

### Scanning electron microscopy

Adult flies were anaesthetized with CO_2_, placed in 25% ethanol for 24 h at room temperature and dehydrated in a graded ethanol series for 3 days. Samples were dried from 100% ethanol with a critical point dryer (EM-CPD300, Leica Microsystems), gold-coated using a sputter coater (EM-ACE600, Leica Microsystems) and imaged at a magnification of 152X (FE-SEM Merlin compact VP, Carl Zeiss; operated at 3 kV).

### Quantification of rough retina phenotype

Three analysis methods were applied to retinas. Fused retinal area was defined as the ratio of the total retina area divided by the total disrupted area. The disrupted area was defined as a region containing two or more fused ommatidia. The number of distinct structures was determined using a distortion algorithm [98]. The output of the algorithm is mapping of boundaries surrounding single or fused ommatidia that are the structures of interest. Structure count and structure centers were then identified in Fiji. Regularity was determined based on structure centers and their nearest neighbor distance distributions using macros written in Igor Pro (Wavemetrics). Regularity was defined as the ratio of the mean nearest neighbor distance and its SD for each image [60].

### Transmission electron microscopy

Thoraces were fixed in 2.5% glutaraldehyde and 2% paraformaldehyde in 0.1 M phosphate buffer (pH 7.4) for 2 h at room temperature. Samples were post-fixed and contrast enhanced with 1% osmium tetroxide in phosphate buffer for 1.5 h and 1% uranyl acetate in 50% ethanol/water for 45 min. Samples were then dehydrated in a graded ethanol series and embedded in Durcupan (Sigma-Aldrich). Ultrathin sections (70 nm) were sliced using a microtome (EM UC7 Ultramicrotome, Leica Microsystems) and mounted on formvar coated copper slot grids. Images were acquired at a magnification of 9000X (Tecnai 10, FEI/Thermo Fisher Scientific; operated at 80 kV, equipped with OSIS Megaview III camera). The electron dense fraction of the cytoplasm (mitochondria) was determined by manual selection, application of threshold and the area fraction command in Fiji.

### ATP determination

Thoraces from two-day old flies were homogenized in 50 μl of extraction buffer (100 mM Tris-HCl, 4 mM EDTA, pH 7.8) supplemented with 6 M Guanidine-HCl using a pellet homogenizer (47747–370, VWR international). The lysate was boiled for 3 min and cleared by centrifugation at 20’000 g for 5 min. The samples were diluted 1:100 in extraction buffer before quantification using a luciferase-based ATP kit (A22066, Thermo Fisher Scientific). Values were expressed relative to total protein concentration measured by using a BCA assay (Pierce). Luminescence and absorbance at 562 nm were measured using the microplate reader. ATP levels were normalized to those of *Opto-dRET* female flies.

### Negative geotaxis (climbing) assay

Male flies of the indicated genotype have been exposed to blue light (I = 320 μW/cm^2^, λ = 470 nm) or kept in the dark during the indicated developmental time points. For each experiment, males hatching on the same day were pooled. Adults were aged for 2–3 days on standard fly food. On day 3, flies were anaesthetized briefly with CO_2_ and 10 flies each were placed in an acrylic glass tube of 30 cm length closed with a flyplug (Carl Roth PK13.1). Flies were allowed to recover and adapt for 1 h. Negative geotaxis climbing performance was then assayed as previously described [99]. Flies were tapped down and the number of flies reaching the 15 cm mark within 15 s was recorded. 10 technical repeats (1 min break between repeats) were performed for each genotype to obtain an average climbing score (defined as fly count above the 15 cm line / total fly count). For each genotype and condition, at least 5 independent experiments were performed.

### Statistical analysis

HEK293 cell assays were performed in triplicate wells and in at least three independent experiments. Statistical analysis (after testing for normality using the Shapiro–Wilk test) was performed using unpaired, two-tailed t-tests for comparison of dark and light conditions.

Fly assays were performed in at least three independent experiments with the number of flies indicated in the figures. Statistical analysis of numerical outcomes (after testing for normality using the Shapiro–Wilk test) was performed using one-way analysis of variance (ANOVA) followed by Bonferroni corrected multiple t-test comparisons. In box plots, the horizontal line, the bottom/top edges of each box, and the vertical lines indicate the median, the 25^th^/75^th^ percentile, and 10^th^/90^th^ percentile, respectively. For categorical outcomes (thorax defect and climbing pass), SEMs shown in the figure derived from the number of flies tested and binomial distributions. Statistical significance was tested using Fisher’s exact tests. Climbing experiments were performed in 10 repeats for each animal group consisting of 10 animals. Statistical significance is indicated using the ‘compact letter display’.

SH-SY5Y cell fragmentation assays were performed in five independent experiments with at least 150 cells per condition in each experiment. Statistical analysis (after testing for normality using the Shapiro–Wilk test) was performed using one-way analysis of variance (ANOVA) followed by followed by Bonferroni corrected multiple t-test comparisons. Statistical significance is indicated using ‘compact letter display’.

Numerical data underlying graphs are provided in S5 Table.

## Supporting information

S1 Fig AU1-LOV is a dimerizing photoreceptor. Upon photoactivation, AU1-LOV associates in a dimeric ‘lit’ state (the star denotes the photoadduct state) [47]. Representations of crystal structures obtained for monomeric and dimeric states of AU1-LOV (PDB-IDs: 5DKK and 5DKL; P. *tricornutum*) [100]. The lit state AU1-LOV domain relaxes to the dark state with a characteristic lifetime τ ~ 600 s [15,101].
(TIF)

S2 Fig Comparison of RET receptors fused to AU1-LOV or FKBP. Receptor activation in response to blue light stimulation (I = 250 μW/cm^2^, λ = 470 nm, 8 h continuous) at the indicated intensity for HEK293 cells transfected with Opto-hRET, or in response to chemical stimulation with 10 nM AP20187 (8 h continuous) for HEK293 cells transfected with hRET-FKBP (F36V variant [95]). Normalized light units (LU; mean ± SD, normalized to mock cells) for the MAPK/ERK pathway-specific transcriptional reporter in unstimulated cells (black bars), light-stimulated cells (blue bars) and AP20187-stimulated cells (open bars) are given (n = 9, three independent experiments, t-test, *: p < .0001).
(TIF)

S3 Fig *Opto-dRET* construct for GAL4-dependent expression in *Drosophila*. *Opto-dRET* or *Opto-dRET*^*MEN2B*^ was inserted in a vector that contains five UAS elements, a mini-*white* gene for visualizing transformants and flanking P-element terminal repeats. Purple stars represent the kinase domain substitution (M955T; ATG to ACG) in *Opto-dRET*^*MEN2B*^. N: N-terminus, LBD: extracellular ligand-binding domain, TMD: single-span transmembrane domain, KD: kinase domain, CTD: C-terminal tail domain, LOV: LOV domain, C: C-terminus.
(TIF)

S4 Fig Retina quantification for alternative illumination regime. (A) Time window targeted by light. (B and C) Representative retina SEM images (image B is taken from Fig 2). (D and E) Quantification of rough retina phenotypes as fused area and the number of structures identified (dark data is taken from Fig 2). In D and E, the number of analyzed flies is given (at least three independent experiments) and bars sharing the same label are not significantly different (ANOVA/Bonferroni corrected t-tests of means, p>.04). Continuous light intensity was 385 μW/cm^2^ for the duration shown in A.
(TIF)

S5 Fig Low-light activation of AU1-LOV. (A) Illumination incubators used in fly experiments. (B) Receptor activation in response to blue light stimulation (8 h continuous) at the indicated intensity for HEK293 cells transfected with *Opto-mFGFR1*. Normalized light units (LU; mean ± SD for the MAPK/ERK pathway-specific transcriptional reporter in control cells (black) and illuminated cells (blue) are given (n = 6, three independent experiments, t-test, *: p < .0001).
(TIF)

S6 Fig Light in the absence of Opto-dRET does not rescue thorax defects. Percentage of flies with a degenerate thorax phenotype. Counts ± SE for the indicated number of flies (n) is given. Percentages sharing the same label are not significantly different (Fisher’s exact test, p>.04). Continuous light intensity was 150–300 μW/cm^2^ for the duration shown in Fig 3A.
(TIF)

S7 Fig Light in the absence of Opto-dRET does not rescue locomotion deficits (climbing ability). Counts ± SE for the indicated number of flies (n) is given. Percentages sharing the same label are not significantly different (Fisher’s exact test, p>.04). Continuous light intensity was 320 μW/cm^2^ for the duration shown in Fig 3A.
(TIF)

S8 Fig Light in the absence of Opto-dRET does not rescue ATP levels. ATP content in fly thoraces from the number of analyzed flies given (normalized to the mean for control flies shown as bar 1). Bars sharing the same label are not significantly different (ANOVA/Bonferroni corrected t-tests of means, p>.04). Continuous light intensity was 320 μW/cm^2^ for the duration shown in Fig 3A.
(TIF)

S9 Fig Light acts specifically through Opto-dRET to rescue mitochondrial fragmentation. In the absence of Opto-dRET, no effect of blue light (I = 232 μW/cm^2^, 4h continuous) is observed for cells transfected with control siRNA (compare bars 1 and 2) or *PINK1* siRNA (compare bar 3 of this figure and bar 2 of Fig 5D). Likewise, light did not impact the rescue of fragmentation by GDNF/GFRα1 (compare bar 4 of this figure and bar 3 of Fig 5D) or by Opto-dRET^MEN2B^ (compare bar 5 of this figure and bar 4 of Fig 5D). “M2B” denotes Opto-dRET^MEN2B^. Mean ± SD for five independent experiments is given (>150 cells/condition/experiment). Means sharing the same label are not significantly different (ANOVA/Bonferroni corrected t-tests, p>.04).
(TIF)

S1 Table (Opto-)dRET sequences. Uniprot/Genbank identifiers are given in parentheses.
(DOCX)

S2 Table (Opto-)hRET sequences. Uniprot identifiers are given in parentheses.
(DOCX)

S3 Table hRET-FKBP sequences. Uniprot identifiers are given in parentheses.
(DOCX)

S4 Table Genotypes of flies in this study. (DOCX)

S5 Table Numerical data underlying graphs. (XLSX)
