# Supplementary figures and images for: Optogenetic delivery of trophic signals in a genetic model of Parkinson’s disease
Source: PLoS Genet. 2021 Apr 15;17(4):e1009479. doi: 10.1371/journal.pgen.1009479 (PMC8049241; doi:10.1371/journal.pgen.1009479)

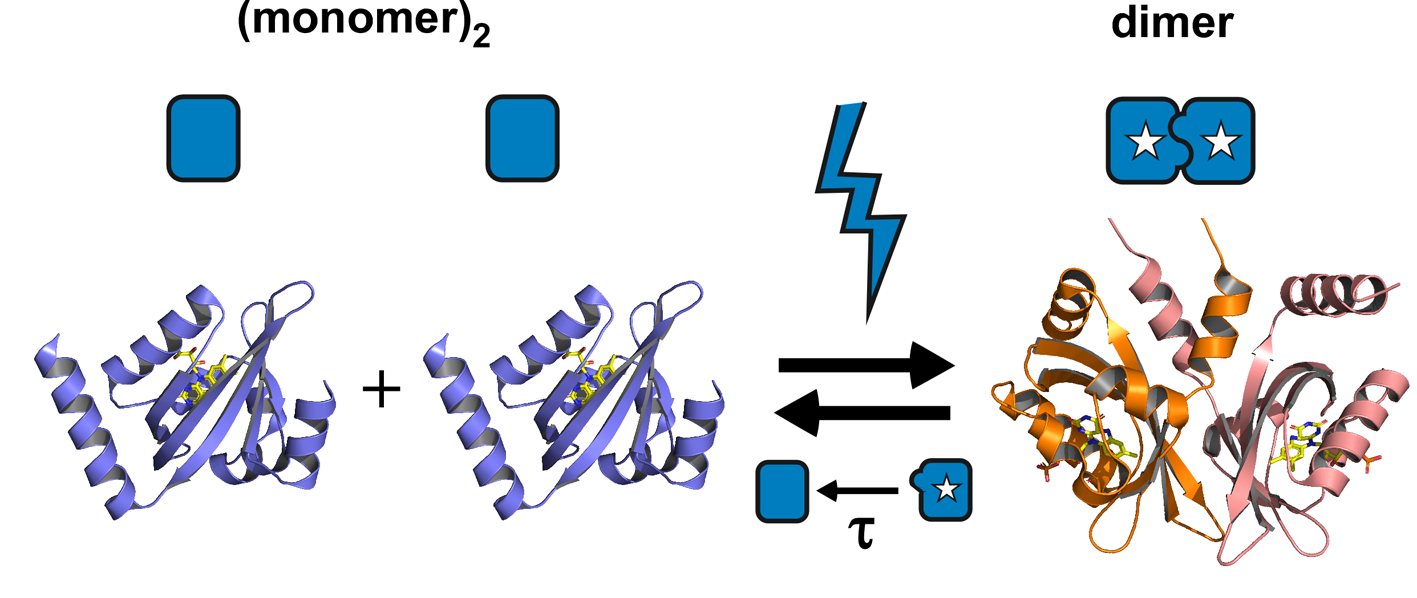

Supplement: S1 Fig — Upon photoactivation, AU1-LOV associates in a dimeric ‘lit’ state (the star denotes the photoadduct state) [47]. Representations of crystal structures obtained for monomeric and dimeric states of AU1-LOV (PDB-IDs: 5DKK and 5DKL; P. tricornutum) [100]. The lit state AU1-LOV domain relaxes to the dark state with a characteristic lifetime τ ~ 600 s [15,101]. (TIF) [file pgen.1009479.s001.tif]

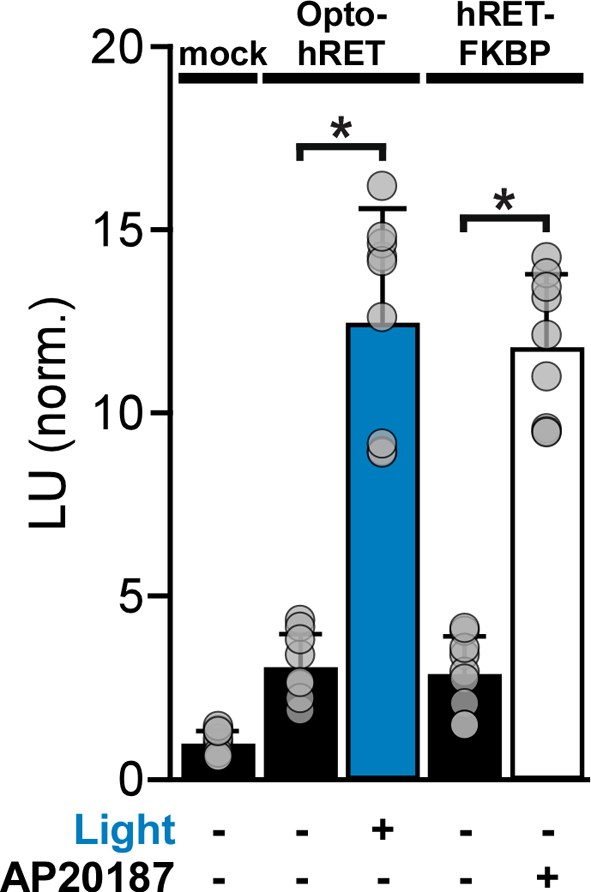

Supplement: S2 Fig — Receptor activation in response to blue light stimulation (I = 250 μW/cm2, λ = 470 nm, 8 h continuous) at the indicated intensity for HEK293 cells transfected with Opto-hRET, or in response to chemical stimulation with 10 nM AP20187 (8 h continuous) for HEK293 cells transfected with hRET-FKBP (F36V variant [95]). Normalized light units (LU; mean ± SD, normalized to mock cells) for the MAPK/ERK pathway-specific transcriptional reporter in unstimulated cells (black bars), light-stimulated cells (blue bars) and AP20187-stimulated cells (open bars) are given (n = 9, three independent experiments, t-test, *: p < .0001). (TIF) [file pgen.1009479.s002.tif]

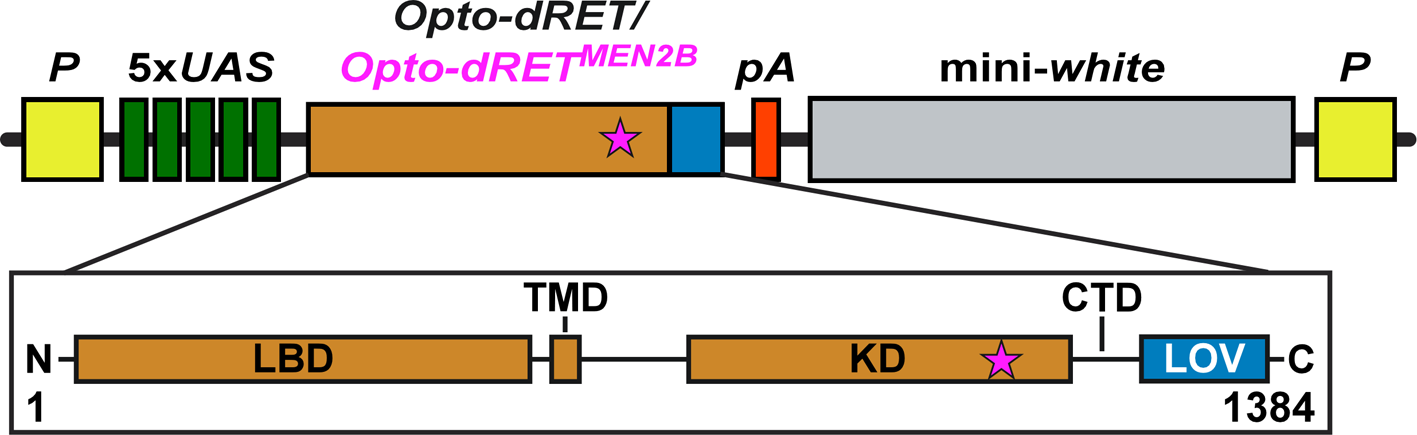

Supplement: S3 Fig — Opto-dRET or Opto-dRETMEN2B was inserted in a vector that contains five UAS elements, a mini-white gene for visualizing transformants and flanking P-element terminal repeats. Purple stars represent the kinase domain substitution (M955T; ATG to ACG) in Opto-dRETMEN2B. N: N-terminus, LBD: extracellular ligand-binding domain, TMD: single-span transmembrane domain, KD: kinase domain, CTD: C-terminal tail domain, LOV: LOV domain, C: C-terminus. (TIF) [file pgen.1009479.s003.tif]

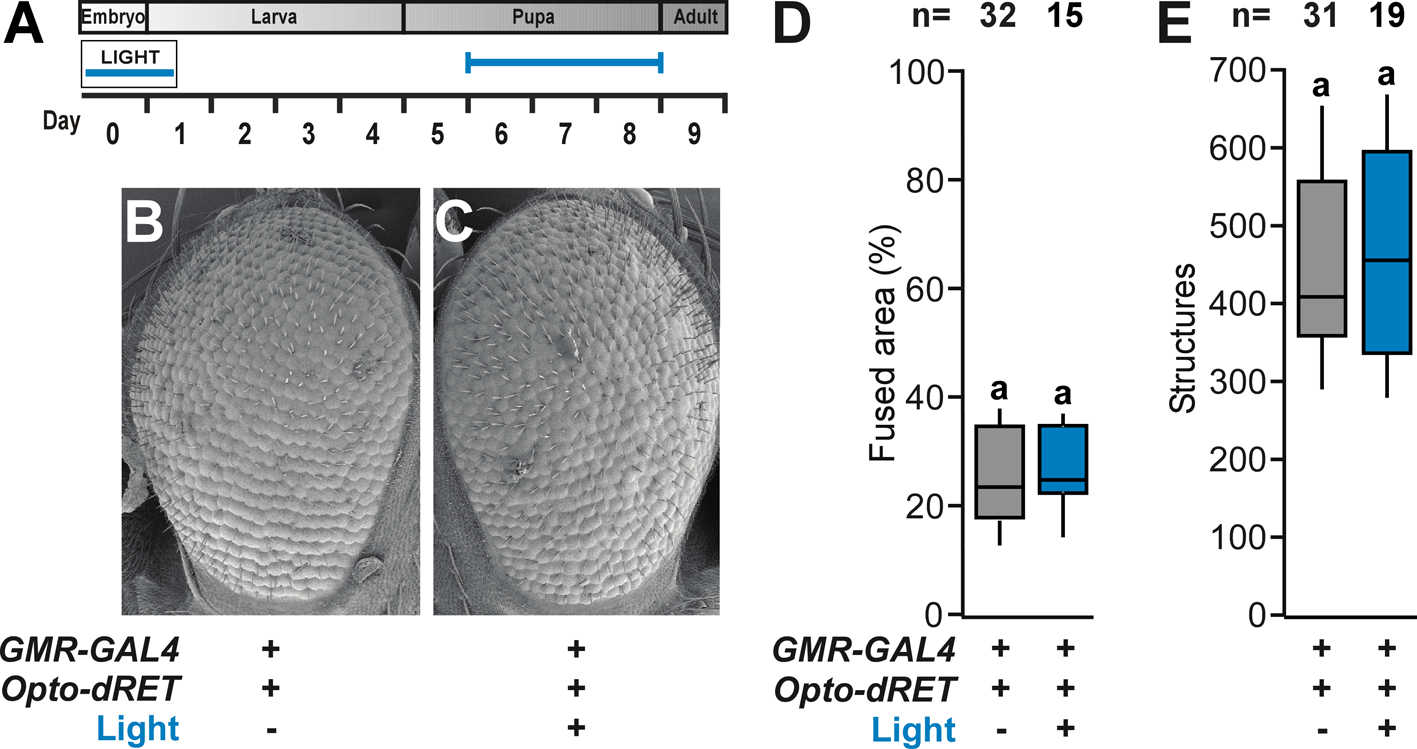

Supplement: S4 Fig — (A) Time window targeted by light. (B and C) Representative retina SEM images (image B is taken from Fig 2). (D and E) Quantification of rough retina phenotypes as fused area and the number of structures identified (dark data is taken from Fig 2). In D and E, the number of analyzed flies is given (at least three independent experiments) and bars sharing the same label are not significantly different (ANOVA/Bonferroni corrected t-tests of means, p>.04). Continuous light intensity was 385 μW/cm2 for the duration shown in A. (TIF) [file pgen.1009479.s004.tif]

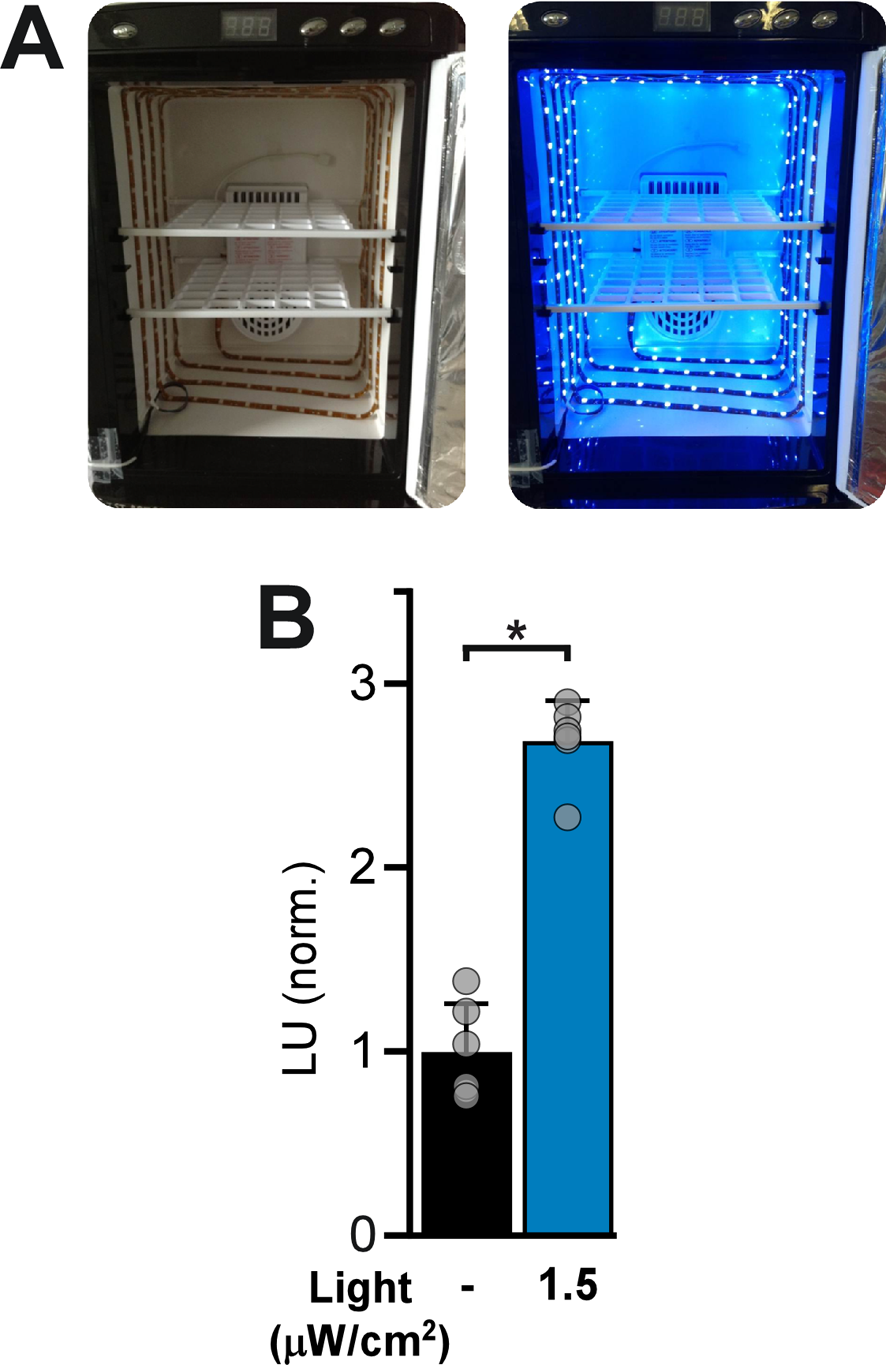

Supplement: S5 Fig — (A) Illumination incubators used in fly experiments. (B) Receptor activation in response to blue light stimulation (8 h continuous) at the indicated intensity for HEK293 cells transfected with Opto-mFGFR1. Normalized light units (LU; mean ± SD for the MAPK/ERK pathway-specific transcriptional reporter in control cells (black) and illuminated cells (blue) are given (n = 6, three independent experiments, t-test, *: p < .0001). (TIF) [file pgen.1009479.s005.tif]

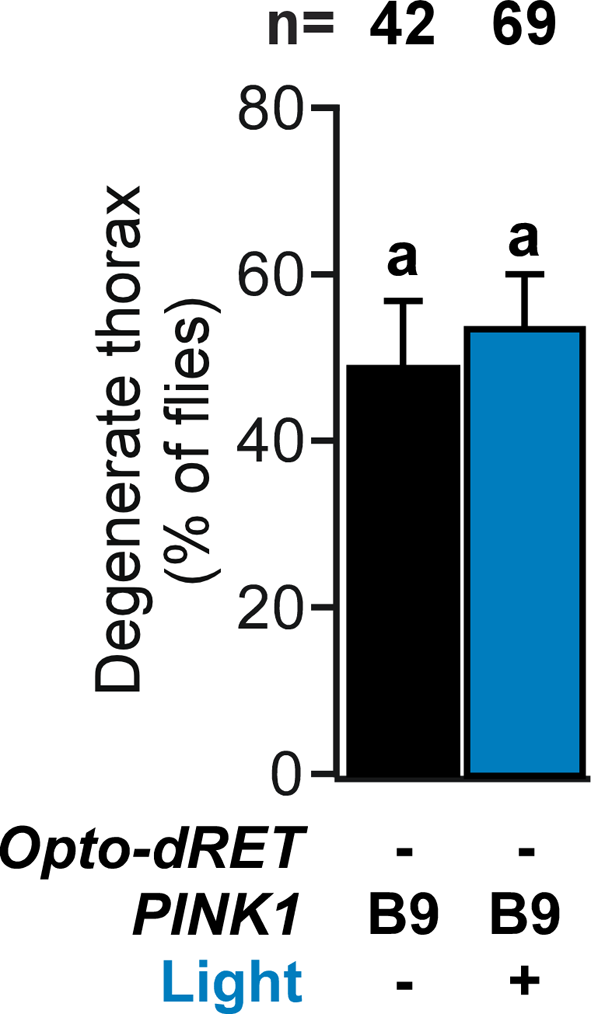

Supplement: S6 Fig — Percentage of flies with a degenerate thorax phenotype. Counts ± SE for the indicated number of flies (n) is given. Percentages sharing the same label are not significantly different (Fisher’s exact test, p>.04). Continuous light intensity was 150–300 μW/cm2 for the duration shown in Fig 3A. (TIF) [file pgen.1009479.s006.tif]

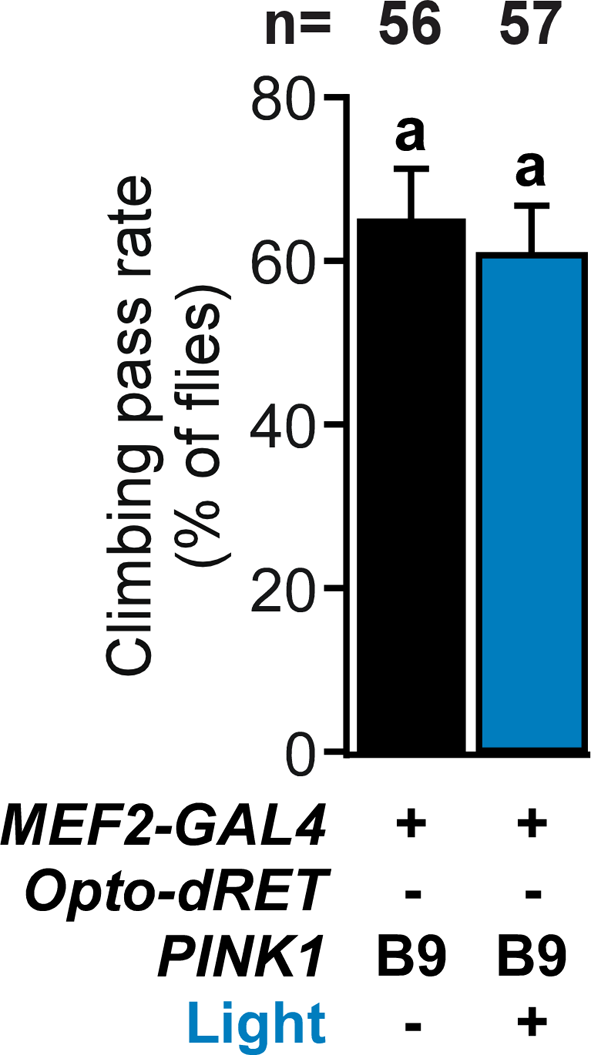

Supplement: S7 Fig — Counts ± SE for the indicated number of flies (n) is given. Percentages sharing the same label are not significantly different (Fisher’s exact test, p>.04). Continuous light intensity was 320 μW/cm2 for the duration shown in Fig 3A. (TIF) [file pgen.1009479.s007.tif]

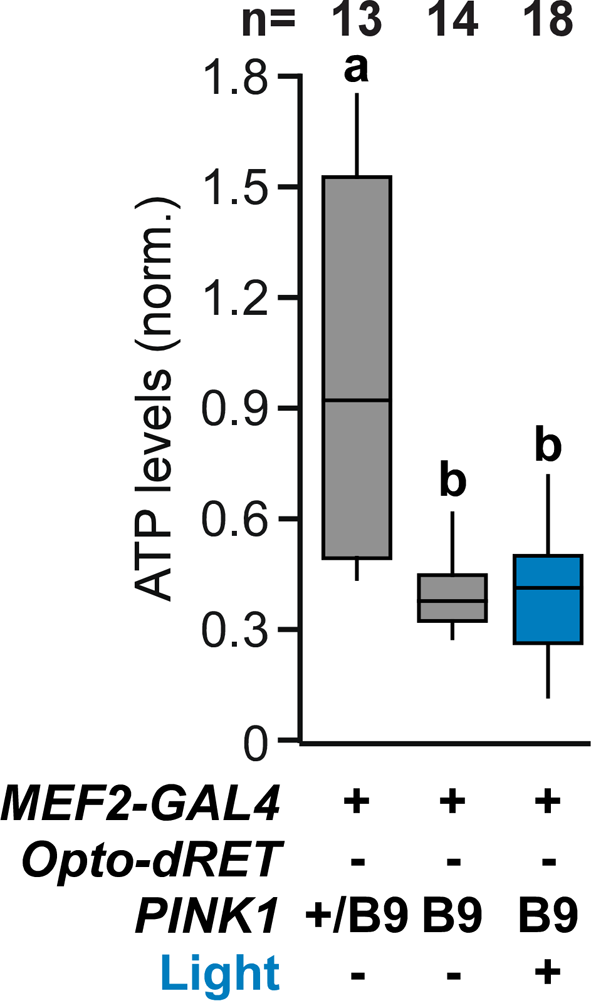

Supplement: S8 Fig — ATP content in fly thoraces from the number of analyzed flies given (normalized to the mean for control flies shown as bar 1). Bars sharing the same label are not significantly different (ANOVA/Bonferroni corrected t-tests of means, p>.04). Continuous light intensity was 320 μW/cm2 for the duration shown in Fig 3A. (TIF) [file pgen.1009479.s008.tif]

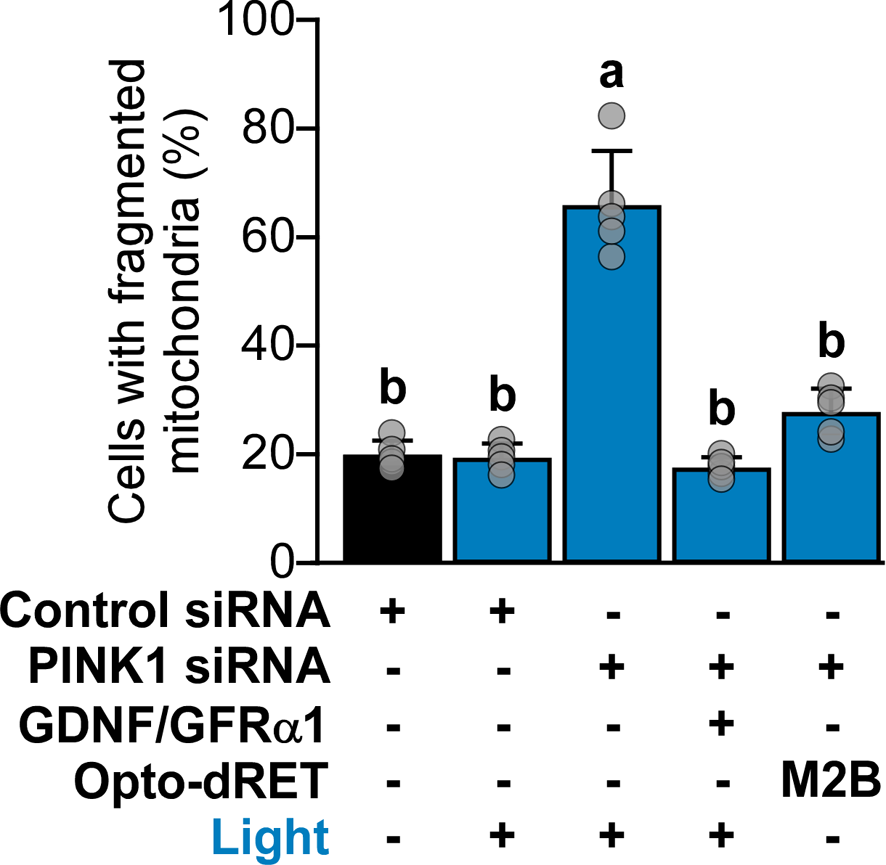

Supplement: S9 Fig — In the absence of Opto-dRET, no effect of blue light (I = 232 μW/cm2, 4h continuous) is observed for cells transfected with control siRNA (compare bars 1 and 2) or PINK1 siRNA (compare bar 3 of this figure and bar 2 of Fig 5D). Likewise, light did not impact the rescue of fragmentation by GDNF/GFRα1 (compare bar 4 of this figure and bar 3 of Fig 5D) or by Opto-dRETMEN2B (compare bar 5 of this figure and bar 4 of Fig 5D). “M2B” denotes Opto-dRETMEN2B. Mean ± SD for five independent experiments is given (>150 cells/condition/experiment). Means sharing the same label are not significantly different (ANOVA/Bonferroni corrected t-tests, p>.04). (TIF) [file pgen.1009479.s009.tif]
