# Supplementary material for: Optogenetic delivery of trophic signals in a genetic model of Parkinson’s disease
Source: PLoS Genet. 2021 Apr 15;17(4):e1009479. doi: 10.1371/journal.pgen.1009479 (PMC8049241; doi:10.1371/journal.pgen.1009479)
Supplement: S1 Table — Uniprot/Genbank identifiers are given in parentheses. (DOCX) [file pgen.1009479.s010.docx]

| **Name** | **Sequence** |
| --- | --- |
| Opto-dRET  (*Drosophila* construct) | MESTTIVFVTLLTIITQRKHCAAVDVYFPTTSVKFNLPINEESESIFSKIPLAQFQVLRMEDNRLASDYLYSLEQNPLLRINSSSGEIYMRTDYRSPNSSATFLVTAFPRDQPDHELLNVSHLSLEVTPQPLEEYCSELEHICFWSSAQYTIAESHGPYRRKDFFEPVLIGALNSRAAKYLCPHVSLEYSLNAGSSHFVLKQNRLYTRQTLDHDELNGLNAKAGQLQARITCTVKLSSRDQRKFSRILDIKLLDRNDNGPKLQESSSKFDFYLEQPYFQADEEAGKKVIYVDKDTLEANAHLVYAVHNDSHGLFRPDCHAYEADHTGRPHTIVSCQLRFSRNGVFRETPYCVSLEARDLTIVSRVDAMSATANVCYHINLSKLHESEQELPQALPLRARQHRIFESEEFNGDSAGRSLSPPTVDYDKDVSVYRSAASNFRVVQPDSFLDLMRLRSIRFDIVEDKLGAFGITSTSGIVFVKNPQVLEEAPETIYFLNVTWIDQQRLSHVRVINVHLVHGRPENTSCELKVKSRSQTCAQIKYQSQCVRYCGLATGGGSCQWRGSNSAMFGTRYGSCVPESRYCPDHVCDPLEELNPMACPQDCTPAGRIVGPHSSNENKRGIYSASGTCICEDNGKCSCAPLDEEPKMKKPRKRKNETEAEPLLGVRRGTPPNQPLQDPMLLGVLNVAGFECDRSCMFFVITCPLLFVLLLLCLLIAQRKMLQRRLGKQSMTTSSKQALPESGGGDFALMPLQSGFRFESGDAKWEFPREKLQLDTVLGEGEFGQVLKGFATEIAGLPGITTVAVKMLKKGSNSVEYMALLSEFQLLQEVSHPNVIKLLGACTSSEAPLLIIEYARYGSLRSYLRLSRKIECAGVDFADGVEPVNVKMVLTFAWQICKGMAYLSELKLVHRDLAARNVLLADGKICKISDFGLTRDVYEDDAYLKRSRDRVPVKWMAPESLADHVYTSKSDVWSFGVLCWELITLGASPYPGIAPQNLWSLLKTGYRMDRPENCSEAVYSIVRTCWADEPNGRPSFKFLASEFEKLLGNNAKYIDLETNAVSNPLYCGDDSALITTELGEPESLQHLWSPPKIAYDIHDQATSYDQSEEEMPVTSTAPPGYDLPRPLLDATANGQVLRYENDLRFPLNIRKSSCTPSYSNMTSEPPATTSLPHYSVPVKRGRSYLDMTNKSLIPDNLDSREFEKHLSKTISFRFSSLLNLSETKEVSPGWQAEDAVTGGPDYSLVKALQMAQQNFVITDASLPDNPIVYASRGFLTLTGYSLDQILGRNCRFLQGPETDPRAVDKIRNAITKGVDTSVCLLNYRQDGTTFWNLFFVAGLRDSKGNIVNYVGVQSKVSEDYAKLLVNEQNIEYKGVRTSNMLRRKPG |
| dRET (CAB96180) | MESTTIVFVTLLTIITQRKHCAAVDVYFPTTSVKFNLPINEESESIFSKIPLAQFQVLRMEDNRLASDYLYSLEQNPLLRINSSSGEIYMRTDYRSPNSSATFLVTAFPRDQPDHELLNVSHLSLEVTPQPLEEYCSELEHICFWSSAQYTIAESHGPYRRKDFFEPVLIGALNSRAAKYLCPHVSLEYSLNAGSSHFVLKQNRLYTRQTLDHDELNGLNAKAGQLQARITCTVKLSSRDQRKFSRILDIKLLDRNDNGPKLQESSSKFDFYLEQPYFQADEEAGKKVIYVDKDTLEANAHLVYAVHNDSHGLFRPDCHAYEADHTGRPHTIVSCQLRFSRNGVFRETPYCVSLEARDLTIVSRVDAMSATANVCYHINLSKLHESEQELPQALPLRARQHRIFESEEFNGDSAGRSLSPPTVDYDKDVSVYRSAASNFRVVQPDSFLDLMRLRSIRFDIVEDKLGAFGITSTSGIVFVKNPQVLEEAPETIYFLNVTWIDQQRLSHVRVINVHLVHGRPENTSCELKVKSRSQTCAQIKYQSQCVRYCGLATGGGSCQWRGSNSAMFGTRYGSCVPESRYCPDHVCDPLEELNPMACPQDCTPAGRIVGPHSSNENKRGIYSASGTCICEDNGKCSCAPLDEEPKMKKPRKRKNETEAEPLLGVRRGTPPNQPLQDPMLLGVLNVAGFECDRSCMFFVITCPLLFVLLLLCLLIAQRKMLQRRLGKQSMTTSSKQALPESGGGDFALMPLQSGFRFESGDAKWEFPREKLQLDTVLGEGEFGQVLKGFATEIAGLPGITTVAVKMLKKGSNSVEYMALLSEFQLLQEVSHPNVIKLLGACTSSEAPLLIIEYARYGSLRSYLRLSRKIECAGVDFADGVEPVNVKMVLTFAWQICKGMAYLSELKLVHRDLAARNVLLADGKICKISDFGLTRDVYEDDAYLKRSRDRVPVKWMAPESLADHVYTSKSDVWSFGVLCWELITLGASPYPGIAPQNLWSLLKTGYRMDRPENCSEAVYSIVRTCWADEPNGRPSFKFLASEFEKLLGNNAKYIDLETNAVSNPLYCGDDSALITTELGEPESLQHLWSPPKIAYDIHDQATSYDQSEEEMPVTSTAPPGYDLPRPLLDATANGQVLRYENDLRFPLNIRKSSCTPSYSNMTSEPPATTSLPHYSVPVKRGRSYLDMTNKSLIPDNLDSREFEKHLSKTISFRFSSLLNLSETKEVSPGWQAEDAV |
| LOV domain of *V. frigida* AUREOCHROME1 (residues 204 to 348 of A8QW55) (AU1-LOV) | PDYSLVKALQMAQQNFVITDASLPDNPIVYASRGFLTLTGYSLDQILGRNCRFLQGPETDPRAVDKIRNAITKGVDTSVCLLNYRQDGTTFWNLFFVAGLRDSKGNIVNYVGVQSKVSEDYAKLLVNEQNIEYKGVRTSNMLRRK |
| *Opto-dRET* (cDNA)  (Drosophila construct) | ATGGAGTCAACTACTATTGTTTTTGTGACTCTGCTCACAATTATAACCCAACGTAAACACTGTGCGGCCGTCGATGTTTACTTTCCCACCACGTCGGTGAAATTCAATCTGCCCATCAATGAGGAATCGGAGAGCATATTCTCCAAAATCCCGCTAGCCCAGTTCCAAGTGCTGCGGATGGAGGACAATCGGTTAGCCAGTGATTACTTATATAGCCTGGAGCAGAATCCACTACTTCGAATAAACAGTTCCTCCGGCGAGATATATATGCGCACTGACTACCGCTCACCAAACTCAAGTGCCACATTCTTGGTGACCGCATTTCCCAGAGATCAACCGGATCACGAGCTGCTGAATGTTTCGCATCTTTCGTTGGAGGTTACACCTCAGCCCCTGGAGGAGTACTGTTCGGAACTGGAGCACATTTGCTTCTGGAGCAGTGCTCAGTACACTATAGCAGAGTCGCACGGTCCATATCGGCGGAAGGATTTTTTTGAACCCGTACTTATCGGCGCCCTTAATTCCCGCGCTGCGAAGTATCTGTGTCCTCATGTATCCCTGGAATACTCCCTGAACGCTGGTAGTTCCCACTTTGTTTTGAAACAAAATCGACTCTACACCCGACAAACCTTGGATCACGACGAGCTCAATGGACTGAATGCCAAGGCAGGGCAGCTGCAGGCCAGGATTACCTGCACGGTTAAATTGTCCAGCAGGGATCAGAGAAAATTCTCGCGCATCTTGGATATCAAGTTACTGGATCGCAATGATAATGGACCCAAGTTGCAGGAGAGTAGCTCTAAGTTTGATTTCTATCTGGAGCAGCCCTACTTCCAAGCGGACGAGGAGGCGGGAAAAAAAGTAATCTACGTGGACAAGGATACATTGGAGGCAAATGCTCACCTTGTCTACGCCGTCCACAATGACTCTCATGGTCTGTTTCGGCCCGACTGCCACGCCTACGAGGCGGATCACACGGGCAGACCACATACCATCGTCAGTTGTCAACTGCGATTCTCCCGAAACGGTGTCTTCCGGGAAACCCCCTATTGTGTGTCCTTGGAGGCTCGGGATCTGACCATTGTAAGCCGTGTCGATGCCATGTCAGCGACAGCCAATGTTTGCTATCATATTAATCTGAGTAAGCTTCACGAATCTGAGCAAGAATTACCGCAAGCTCTTCCCCTACGGGCACGTCAACATCGAATATTCGAGAGCGAAGAATTCAATGGAGATTCTGCAGGCCGATCTCTAAGTCCTCCGACCGTGGATTACGATAAGGATGTTTCCGTATACAGATCGGCTGCTTCTAATTTTCGAGTTGTCCAGCCTGACAGTTTTTTGGACTTGATGCGGTTACGATCTATTCGATTCGATATTGTGGAGGATAAACTTGGAGCTTTTGGTATTACCTCAACATCGGGTATTGTCTTTGTGAAGAACCCACAGGTTTTGGAGGAGGCACCGGAAACCATATACTTCCTGAATGTCACCTGGATCGATCAGCAAAGGCTGTCGCACGTGAGAGTGATCAATGTGCACTTGGTTCATGGTAGACCCGAGAATACTAGTTGCGAACTGAAGGTCAAGTCTCGATCACAGACATGTGCCCAGATTAAATACCAATCGCAATGCGTTCGATATTGCGGCTTGGCCACAGGTGGTGGATCTTGCCAGTGGAGGGGGTCCAACTCAGCCATGTTCGGCACTAGATATGGTTCCTGTGTACCCGAATCTCGTTACTGTCCAGATCATGTCTGTGATCCCCTAGAGGAACTGAATCCTATGGCCTGTCCGCAGGATTGCACGCCAGCTGGAAGAATCGTGGGTCCCCATTCAAGTAATGAGAATAAGAGAGGGATATACAGTGCCTCGGGTACCTGCATTTGCGAGGATAATGGCAAGTGCTCGTGCGCTCCGTTAGATGAGGAACCCAAGATGAAGAAACCGCGAAAACGAAAAAACGAAACAGAGGCGGAACCTCTGCTGGGGGTACGAAGGGGCACTCCTCCGAATCAGCCACTTCAGGATCCCATGCTTCTGGGTGTCCTAAATGTGGCCGGTTTCGAATGCGATCGCTCCTGCATGTTCTTCGTGATCACGTGCCCTCTATTGTTCGTTCTCCTGCTCCTCTGTTTGCTGATTGCGCAGAGAAAGATGCTCCAACGTCGCTTGGGCAAGCAATCAATGACCACGTCGTCGAAACAAGCTCTTCCGGAATCAGGAGGCGGAGATTTCGCCTTGATGCCGCTGCAGAGTGGCTTCAGGTTCGAAAGTGGGGATGCCAAATGGGAGTTTCCCAGGGAAAAACTGCAACTAGATACGGTCTTGGGAGAGGGTGAATTTGGTCAGGTGCTAAAGGGCTTTGCCACCGAGATCGCTGGCTTGCCGGGAATAACCACGGTGGCCGTAAAGATGCTCAAGAAGGGCTCCAATTCAGTGGAGTACATGGCCCTGCTTTCGGAGTTTCAGCTCCTCCAGGAGGTCTCTCACCCGAATGTGATCAAGCTGCTAGGCGCCTGCACCTCCTCCGAAGCACCTCTCCTGATCATCGAGTATGCTCGGTATGGCTCTCTGAGGAGCTATCTTCGACTCAGTCGGAAGATCGAGTGTGCCGGCGTAGATTTCGCAGATGGAGTGGAGCCTGTTAATGTTAAGATGGTACTTACCTTTGCTTGGCAGATTTGCAAGGGTATGGCTTACCTTTCTGAGTTAAAGTTGGTTCATCGTGATTTGGCTGCTAGAAATGTGCTCCTTGCGGATGGCAAGATATGCAAAATATCAGATTTCGGACTGACTCGAGATGTTTACGAGGACGATGCCTATTTAAAGAGATCCCGAGATCGTGTGCCCGTCAAGTGGATGGCTCCGGAATCTTTAGCGGATCATGTGTATACCAGCAAATCGGATGTGTGGTCCTTTGGCGTTCTCTGCTGGGAACTAATCACTCTGGGAGCCTCTCCGTATCCTGGCATTGCTCCCCAGAATTTGTGGTCCTTGCTGAAGACGGGCTACCGCATGGACAGACCAGAAAACTGTTCGGAGGCTGTCTACTCTATAGTTCGAACTTGCTGGGCAGACGAGCCAAATGGAAGACCCTCATTCAAGTTTTTAGCATCGGAGTTTGAGAAGCTATTGGGAAACAATGCCAAGTACATAGATCTGGAAACGAATGCCGTTTCGAATCCCCTTTATTGTGGGGATGATTCCGCCTTAATAACCACGGAATTGGGCGAACCAGAATCGTTGCAGCACCTTTGGTCACCTCCCAAAATAGCCTACGACATCCATGACCAGGCCACCAGCTACGATCAGTCTGAGGAGGAGATGCCAGTGACTTCAACGGCTCCGCCGGGTTACGACTTACCACGACCTTTGCTTGATGCTACCGCCAACGGGCAGGTTTTGCGATACGAAAACGATTTGCGATTCCCCTTAAATATTCGGAAATCCAGTTGTACTCCAAGCTACAGCAACATGACCAGTGAACCTCCAGCGACCACCTCACTGCCACATTATTCTGTCCCCGTGAAGAGGGGTCGATCCTACCTGGATATGACCAACAAGAGTCTCATCCCAGACAACCTGGACAGCAGGGAGTTTGAAAAGCATCTGTCCAAGACCATCTCGTTCCGTTTCTCTAGTTTGCTGAATCTCAGTGAAACGAAGGAGGTGAGTCCAGGATGGCAAGCTGAGGATGCAGTCACCGGTGGACCTGACTACAGTCTCGTGAAGGCTCTGCAAATGGCACAACAGAATTTTGTCATTACAGACGCCTCCCTCCCAGACAACCCTATCGTCTACGCCAGTAGAGGGTTTCTGACACTGACAGGCTATTCTCTCGACCAGATCCTGGGCAGGAACTGCAGGTTTCTGCAAGGGCCAGAAACAGACCCAAGAGCTGTGGATAAGATCAGGAATGCCATCACCAAAGGCGTTGATACCAGTGTCTGTCTGCTGAATTATAGACAGGATGGCACAACCTTCTGGAATCTCTTCTTCGTGGCTGGACTCAGAGATTCTAAGGGCAATATTGTCAACTACGTCGGAGTGCAGTCAAAGGTGAGCGAAGATTATGCCAAGCTGCTGGTCAACGAGCAGAACATTGAGTACAAAGGTGTGCGCACCAGTAACATGCTGCGCAGAAAGCCCGGTTAG |
