# Supplementary material for: Optogenetic delivery of trophic signals in a genetic model of Parkinson’s disease
Source: PLoS Genet. 2021 Apr 15;17(4):e1009479. doi: 10.1371/journal.pgen.1009479 (PMC8049241; doi:10.1371/journal.pgen.1009479)
Supplement: S2 Table — Uniprot identifiers are given in parentheses. (DOCX) [file pgen.1009479.s011.docx]

| **Name** | **Sequence** |
| --- | --- |
| Opto-hRET  (Mammalian construct, including HA-tag) | MAKATSGAAGLRLLLLLLLPLLGKVALGLYFSRDAYWEKLYVDQAAGTPLLYVHALRDAPEEVPSFRLGQHLYGTYRTRLHENNWICIQEDTGLLYLNRSLDHSSWEKLSVRNRGFPLLTVYLKVFLSPTSLREGECQWPGCARVYFSFFNTSFPACSSLKPRELCFPETRPSFRIRENRPPGTFHQFRLLPVQFLCPNISVAYRLLEGEGLPFRCAPDSLEVSTRWALDREQREKYELVAVCTVHAGAREEVVMVPFPVTVYDEDDSAPTFPAGVDTASAVVEFKRKEDTVVATLRVFDADVVPASGELVRRYTSTLLPGDTWAQQTFRVEHWPNETSVQANGSFVRATVHDYRLVLNRNLSISENRTMQLAVLVNDSDFQGPGAGVLLLHFNVSVLPVSLHLPSTYSLSVSRRARRFAQIGKVCVENCQAFSGINVQYKLHSSGANCSTLGVVTSAEDTSGILFVNDTKALRRPKCAELHYMVVATDQQTSRQAQAQLLVTVEGSYVAEEAGCPLSCAVSKRRLECEECGGLGSPTGRCEWRQGDGKGITRNFSTCSPSTKTCPDGHCDVVETQDINICPQDCLRGSIVGGHEPGEPRGIKAGYGTCNCFPEEEKCFCEPEDIQDPLCDELCRTVIAAAVLFSFIVSVLLSAFCIHCYHKFAHKPPISSAEMTFRRPAQAFPVSYSSSGARRPSLDSMENQVSVDAFKILEDPKWEFPRKNLVLGKTLGEGEFGKVVKATAFHLKGRAGYTTVAVKMLKENASPSELRDLLSEFNVLKQVNHPHVIKLYGACSQDGPLLLIVEYAKYGSLRGFLRESRKVGPGYLGSGGSRNSSSLDHPDERALTMGDLISFAWQISQGMQYLAEMKLVHRDLAARNILVAEGRKMKISDFGLSRDVYEEDSYVKRSQGRIPVKWMAIESLFDHIYTTQSDVWSFGVLLWEIVTLGGNPYPGIPPERLFNLLKTGHRMERPDNCSEEMYRLMLQCWKQEPDKRPVFADISKDLEKMMVKRRDYLDLAASTPSDSLIYDDGLSEEETPLVDCNNAPLPRALPSTWIENKLYGRISHAFTRFTGGPDYSLVKALQMAQQNFVITDASLPDNPIVYASRGFLTLTGYSLDQILGRNCRFLQGPETDPRAVDKIRNAITKGVDTSVCLLNYRQDGTTFWNLFFVAGLRDSKGNIVNYVGVQSKVSEDYAKLLVNEQNIEYKGVRTSNMLRRKPGGSGVDYPYDVPDYALD |
| hRET (P07949-2) | MAKATSGAAGLRLLLLLLLPLLGKVALGLYFSRDAYWEKLYVDQAAGTPLLYVHALRDAPEEVPSFRLGQHLYGTYRTRLHENNWICIQEDTGLLYLNRSLDHSSWEKLSVRNRGFPLLTVYLKVFLSPTSLREGECQWPGCARVYFSFFNTSFPACSSLKPRELCFPETRPSFRIRENRPPGTFHQFRLLPVQFLCPNISVAYRLLEGEGLPFRCAPDSLEVSTRWALDREQREKYELVAVCTVHAGAREEVVMVPFPVTVYDEDDSAPTFPAGVDTASAVVEFKRKEDTVVATLRVFDADVVPASGELVRRYTSTLLPGDTWAQQTFRVEHWPNETSVQANGSFVRATVHDYRLVLNRNLSISENRTMQLAVLVNDSDFQGPGAGVLLLHFNVSVLPVSLHLPSTYSLSVSRRARRFAQIGKVCVENCQAFSGINVQYKLHSSGANCSTLGVVTSAEDTSGILFVNDTKALRRPKCAELHYMVVATDQQTSRQAQAQLLVTVEGSYVAEEAGCPLSCAVSKRRLECEECGGLGSPTGRCEWRQGDGKGITRNFSTCSPSTKTCPDGHCDVVETQDINICPQDCLRGSIVGGHEPGEPRGIKAGYGTCNCFPEEEKCFCEPEDIQDPLCDELCRTVIAAAVLFSFIVSVLLSAFCIHCYHKFAHKPPISSAEMTFRRPAQAFPVSYSSSGARRPSLDSMENQVSVDAFKILEDPKWEFPRKNLVLGKTLGEGEFGKVVKATAFHLKGRAGYTTVAVKMLKENASPSELRDLLSEFNVLKQVNHPHVIKLYGACSQDGPLLLIVEYAKYGSLRGFLRESRKVGPGYLGSGGSRNSSSLDHPDERALTMGDLISFAWQISQGMQYLAEMKLVHRDLAARNILVAEGRKMKISDFGLSRDVYEEDSYVKRSQGRIPVKWMAIESLFDHIYTTQSDVWSFGVLLWEIVTLGGNPYPGIPPERLFNLLKTGHRMERPDNCSEEMYRLMLQCWKQEPDKRPVFADISKDLEKMMVKRRDYLDLAASTPSDSLIYDDGLSEEETPLVDCNNAPLPRALPSTWIENKLYGRISHAFTRF |
| *Opto-hRET* (cDNA)  (Mammalian construct, including HA-tag) | ATGGCGAAGGCGACGTCCGGTGCCGCGGGGCTGCGTCTGCTGTTGCTGCTGCTGCTGCCGCTGCTAGGCAAAGTGGCATTGGGCCTCTACTTCTCGAGGGATGCTTACTGGGAGAAGCTGTATGTGGACCAGGCGGCCGGCACGCCCTTGCTGTACGTCCATGCCCTGCGGGACGCCCCTGAGGAGGTGCCCAGCTTCCGCCTGGGCCAGCATCTCTACGGCACGTACCGCACACGGCTGCATGAGAACAACTGGATCTGCATCCAGGAGGACACCGGCCTCCTCTACCTTAACCGGAGCCTGGACCATAGCTCCTGGGAGAAGCTCAGTGTCCGCAACCGCGGCTTTCCCCTGCTCACCGTCTACCTCAAGGTCTTCCTGTCACCCACATCCCTTCGTGAGGGCGAGTGCCAGTGGCCAGGCTGTGCCCGCGTATACTTCTCCTTCTTCAACACCTCCTTTCCAGCCTGCAGCTCCCTCAAGCCCCGGGAGCTCTGCTTCCCAGAGACAAGGCCCTCCTTCCGCATTCGGGAGAACCGACCCCCAGGCACCTTCCACCAGTTCCGCCTGCTGCCTGTGCAGTTCTTGTGCCCCAACATCAGCGTGGCCTACAGGCTCCTGGAGGGTGAGGGTCTGCCCTTCCGCTGCGCCCCGGACAGCCTGGAGGTGAGCACGCGCTGGGCCCTGGACCGCGAGCAGCGGGAGAAGTACGAGCTGGTGGCCGTGTGCACCGTGCACGCCGGCGCGCGCGAGGAGGTGGTGATGGTGCCCTTCCCGGTGACCGTGTACGACGAGGACGACTCGGCGCCCACCTTCCCCGCGGGCGTCGACACCGCCAGCGCCGTGGTGGAGTTCAAGCGGAAGGAGGACACCGTGGTGGCCACGCTGCGTGTCTTCGATGCAGACGTGGTACCTGCATCAGGGGAGCTGGTGAGGCGGTACACAAGCACGCTGCTCCCCGGGGACACCTGGGCCCAGCAGACCTTCCGGGTGGAACACTGGCCCAACGAGACCTCGGTCCAGGCCAACGGCAGCTTCGTGCGGGCGACCGTACATGACTATAGGCTGGTTCTCAACCGGAACCTCTCCATCTCGGAGAACCGCACCATGCAGCTGGCGGTGCTGGTCAATGACTCAGACTTCCAGGGCCCAGGAGCGGGCGTCCTCTTGCTCCACTTCAACGTGTCGGTGCTGCCGGTCAGCCTGCACCTGCCCAGTACCTACTCCCTCTCCGTGAGCAGGAGGGCTCGCCGATTTGCCCAGATCGGGAAAGTCTGTGTGGAAAACTGCCAGGCATTCAGTGGCATCAACGTCCAGTACAAGCTGCATTCCTCTGGTGCCAACTGCAGCACGCTAGGGGTGGTCACCTCAGCCGAGGACACCTCGGGGATCCTGTTTGTGAATGACACCAAGGCCCTGCGGCGGCCCAAGTGTGCCGAACTTCACTACATGGTGGTGGCCACCGACCAGCAGACCTCTAGGCAGGCCCAGGCCCAGCTGCTTGTAACAGTGGAGGGGTCATATGTGGCCGAGGAGGCGGGCTGCCCCCTGTCCTGTGCAGTCAGCAAGAGACGGCTGGAGTGTGAGGAGTGTGGCGGCCTGGGCTCCCCAACAGGCAGGTGTGAGTGGAGGCAAGGAGATGGCAAAGGGATCACCAGGAACTTCTCCACCTGCTCTCCCAGCACCAAGACCTGCCCCGACGGCCACTGCGATGTTGTGGAGACCCAAGACATCAACATTTGCCCTCAGGACTGCCTCCGGGGCAGCATTGTTGGGGGACACGAGCCTGGGGAGCCCCGGGGGATTAAAGCTGGCTATGGCACCTGCAACTGCTTCCCTGAGGAGGAGAAGTGCTTCTGCGAGCCCGAAGACATCCAGGATCCACTGTGCGACGAGCTGTGCCGCACGGTGATCGCAGCCGCTGTCCTCTTCTCCTTCATCGTCTCGGTGCTGCTGTCTGCCTTCTGCATCCACTGCTACCACAAGTTTGCCCACAAGCCACCCATCTCCTCAGCTGAGATGACCTTCCGGAGGCCCGCCCAGGCCTTCCCGGTCAGCTACTCCTCTTCCGGTGCCCGCCGGCCCTCGCTGGACTCCATGGAGAACCAGGTCTCCGTGGATGCCTTCAAGATCCTGGAGGATCCAAAGTGGGAATTCCCTCGGAAGAACTTGGTTCTTGGAAAAACTCTAGGAGAAGGCGAATTTGGAAAAGTGGTCAAGGCAACGGCCTTCCATCTGAAAGGCAGAGCAGGGTACACCACGGTGGCCGTGAAGATGCTGAAAGAGAACGCCTCCCCGAGTGAGCTTCGAGACCTGCTGTCAGAGTTCAACGTCCTGAAGCAGGTCAACCACCCACATGTCATCAAATTGTATGGGGCCTGCAGCCAGGATGGCCCGCTCCTCCTCATCGTGGAGTACGCCAAATACGGCTCCCTGCGGGGCTTCCTCCGCGAGAGCCGCAAAGTGGGGCCTGGCTACCTGGGCAGTGGAGGCAGCCGCAACTCCAGCTCCCTGGACCACCCGGATGAGCGGGCCCTCACCATGGGCGACCTCATCTCATTTGCCTGGCAGATCTCACAGGGGATGCAGTATCTGGCCGAGATGAAGCTCGTTCATCGGGACTTGGCAGCCAGAAACATCCTGGTAGCTGAGGGGCGGAAGATGAAGATTTCGGATTTCGGCTTGTCCCGAGATGTTTATGAAGAGGATTCCTACGTGAAGAGGAGCCAGGGTCGGATTCCAGTTAAATGGATGGCAATTGAATCCCTTTTTGATCATATCTACACCACGCAAAGTGATGTATGGTCTTTTGGTGTCCTGCTGTGGGAGATCGTGACCCTAGGGGGAAACCCCTATCCTGGGATTCCTCCTGAGCGGCTCTTCAACCTTCTGAAGACCGGCCACCGGATGGAGAGGCCAGACAACTGCAGCGAGGAGATGTACCGCCTGATGCTGCAATGCTGGAAGCAGGAGCCGGACAAAAGGCCGGTGTTTGCGGACATCAGCAAAGACCTGGAGAAGATGATGGTTAAGAGGAGAGACTACTTGGACCTTGCGGCGTCCACTCCATCTGACTCCCTGATTTATGACGACGGCCTCTCAGAGGAGGAGACACCGCTGGTGGACTGTAATAATGCCCCCCTCCCTCGAGCCCTCCCTTCCACATGGATTGAAAACAAACTCTATGGTAGAATTTCCCATGCATTTACTAGATTCAccggTGGACCTGACTACAGTCTCGTGAAGGCTCTGCAAATGGCACAACAGAATTTTGTCATTACAGACGCCTCCCTCCCAGACAACCCTATCGTCTACGCCAGTAGAGGGTTTCTGACACTGACAGGCTATTCTCTCGACCAGATCCTGGGCAGGAACTGCAGGTTTCTGCAAGGGCCAGAAACAGACCCAAGAGCTGTGGATAAGATCAGGAATGCCATCACCAAAGGCGTTGATACCAGTGTCTGTCTGCTGAATTATAGACAGGATGGCACAACCTTCTGGAATCTCTTCTTCGTGGCTGGACTCAGAGATTCTAAGGGCAATATTGTCAACTACGTCGGAGTGCAGTCAAAGGTGAGCGAAGATTATGCCAAGCTGCTGGTCAACGAGCAGAACATTGAGTACAAAGGTGTGCGCACCAGTAACATGCTGCGCAGAAAGCCCGGTGGATCCGGAGTCGACTATCCGTACGACGTACCAGACTACGCACTCGACTAA |
