# Supplementary material for: Optogenetic delivery of trophic signals in a genetic model of Parkinson’s disease
Source: PLoS Genet. 2021 Apr 15;17(4):e1009479. doi: 10.1371/journal.pgen.1009479 (PMC8049241; doi:10.1371/journal.pgen.1009479)
Supplement: S3 Table — Uniprot identifiers are given in parentheses. (DOCX) [file pgen.1009479.s012.docx]

| **Name** | **Sequence** |
| --- | --- |
| hRET-FKBP  (Mammalian construct, including HA-tag) | MAKATSGAAGLRLLLLLLLPLLGKVALGLYFSRDAYWEKLYVDQAAGTPLLYVHALRDAPEEVPSFRLGQHLYGTYRTRLHENNWICIQEDTGLLYLNRSLDHSSWEKLSVRNRGFPLLTVYLKVFLSPTSLREGECQWPGCARVYFSFFNTSFPACSSLKPRELCFPETRPSFRIRENRPPGTFHQFRLLPVQFLCPNISVAYRLLEGEGLPFRCAPDSLEVSTRWALDREQREKYELVAVCTVHAGAREEVVMVPFPVTVYDEDDSAPTFPAGVDTASAVVEFKRKEDTVVATLRVFDADVVPASGELVRRYTSTLLPGDTWAQQTFRVEHWPNETSVQANGSFVRATVHDYRLVLNRNLSISENRTMQLAVLVNDSDFQGPGAGVLLLHFNVSVLPVSLHLPSTYSLSVSRRARRFAQIGKVCVENCQAFSGINVQYKLHSSGANCSTLGVVTSAEDTSGILFVNDTKALRRPKCAELHYMVVATDQQTSRQAQAQLLVTVEGSYVAEEAGCPLSCAVSKRRLECEECGGLGSPTGRCEWRQGDGKGITRNFSTCSPSTKTCPDGHCDVVETQDINICPQDCLRGSIVGGHEPGEPRGIKAGYGTCNCFPEEEKCFCEPEDIQDPLCDELCRTVIAAAVLFSFIVSVLLSAFCIHCYHKFAHKPPISSAEMTFRRPAQAFPVSYSSSGARRPSLDSMENQVSVDAFKILEDPKWEFPRKNLVLGKTLGEGEFGKVVKATAFHLKGRAGYTTVAVKMLKENASPSELRDLLSEFNVLKQVNHPHVIKLYGACSQDGPLLLIVEYAKYGSLRGFLRESRKVGPGYLGSGGSRNSSSLDHPDERALTMGDLISFAWQISQGMQYLAEMKLVHRDLAARNILVAEGRKMKISDFGLSRDVYEEDSYVKRSQGRIPVKWMAIESLFDHIYTTQSDVWSFGVLLWEIVTLGGNPYPGIPPERLFNLLKTGHRMERPDNCSEEMYRLMLQCWKQEPDKRPVFADISKDLEKMMVKRRDYLDLAASTPSDSLIYDDGLSEEETPLVDCNNAPLPRALPSTWIENKLYGRISHAFTRFTGGKLEVEGVQVETISPGDGRTFPKRGQTCVVHYTGMLEDGKKVDSSRDRNKPFKFMLGKQEVIRGWEEGVAQMSVGQRAKLTISPDYAYGATGHPGIIPPHATLVFDVELLKLESGGTGGSGVDYPYDVPDYALD |
| FKBP (residues 2 to 108 of P62942 with optimizing F36V substitution [[5](#_ENREF_5)]) | GVQVETISPGDGRTFPKRGQTCVVHYTGMLEDGKKVDSSRDRNKPFKFMLGKQEVIRGWEEGVAQMSVGQRAKLTISPDYAYGATGHPGIIPPHATLVFDVELLKL |
| *hRET-FKBP* (cDNA)  (Mammalian construct, including HA-tag) | ATGGCGAAGGCGACGTCCGGTGCCGCGGGGCTGCGTCTGCTGTTGCTGCTGCTGCTGCCGCTGCTAGGCAAAGTGGCATTGGGCCTCTACTTCTCGAGGGATGCTTACTGGGAGAAGCTGTATGTGGACCAGGCGGCCGGCACGCCCTTGCTGTACGTCCATGCCCTGCGGGACGCCCCTGAGGAGGTGCCCAGCTTCCGCCTGGGCCAGCATCTCTACGGCACGTACCGCACACGGCTGCATGAGAACAACTGGATCTGCATCCAGGAGGACACCGGCCTCCTCTACCTTAACCGGAGCCTGGACCATAGCTCCTGGGAGAAGCTCAGTGTCCGCAACCGCGGCTTTCCCCTGCTCACCGTCTACCTCAAGGTCTTCCTGTCACCCACATCCCTTCGTGAGGGCGAGTGCCAGTGGCCAGGCTGTGCCCGCGTATACTTCTCCTTCTTCAACACCTCCTTTCCAGCCTGCAGCTCCCTCAAGCCCCGGGAGCTCTGCTTCCCAGAGACAAGGCCCTCCTTCCGCATTCGGGAGAACCGACCCCCAGGCACCTTCCACCAGTTCCGCCTGCTGCCTGTGCAGTTCTTGTGCCCCAACATCAGCGTGGCCTACAGGCTCCTGGAGGGTGAGGGTCTGCCCTTCCGCTGCGCCCCGGACAGCCTGGAGGTGAGCACGCGCTGGGCCCTGGACCGCGAGCAGCGGGAGAAGTACGAGCTGGTGGCCGTGTGCACCGTGCACGCCGGCGCGCGCGAGGAGGTGGTGATGGTGCCCTTCCCGGTGACCGTGTACGACGAGGACGACTCGGCGCCCACCTTCCCCGCGGGCGTCGACACCGCCAGCGCCGTGGTGGAGTTCAAGCGGAAGGAGGACACCGTGGTGGCCACGCTGCGTGTCTTCGATGCAGACGTGGTACCTGCATCAGGGGAGCTGGTGAGGCGGTACACAAGCACGCTGCTCCCCGGGGACACCTGGGCCCAGCAGACCTTCCGGGTGGAACACTGGCCCAACGAGACCTCGGTCCAGGCCAACGGCAGCTTCGTGCGGGCGACCGTACATGACTATAGGCTGGTTCTCAACCGGAACCTCTCCATCTCGGAGAACCGCACCATGCAGCTGGCGGTGCTGGTCAATGACTCAGACTTCCAGGGCCCAGGAGCGGGCGTCCTCTTGCTCCACTTCAACGTGTCGGTGCTGCCGGTCAGCCTGCACCTGCCCAGTACCTACTCCCTCTCCGTGAGCAGGAGGGCTCGCCGATTTGCCCAGATCGGGAAAGTCTGTGTGGAAAACTGCCAGGCATTCAGTGGCATCAACGTCCAGTACAAGCTGCATTCCTCTGGTGCCAACTGCAGCACGCTAGGGGTGGTCACCTCAGCCGAGGACACCTCGGGGATCCTGTTTGTGAATGACACCAAGGCCCTGCGGCGGCCCAAGTGTGCCGAACTTCACTACATGGTGGTGGCCACCGACCAGCAGACCTCTAGGCAGGCCCAGGCCCAGCTGCTTGTAACAGTGGAGGGGTCATATGTGGCCGAGGAGGCGGGCTGCCCCCTGTCCTGTGCAGTCAGCAAGAGACGGCTGGAGTGTGAGGAGTGTGGCGGCCTGGGCTCCCCAACAGGCAGGTGTGAGTGGAGGCAAGGAGATGGCAAAGGGATCACCAGGAACTTCTCCACCTGCTCTCCCAGCACCAAGACCTGCCCCGACGGCCACTGCGATGTTGTGGAGACCCAAGACATCAACATTTGCCCTCAGGACTGCCTCCGGGGCAGCATTGTTGGGGGACACGAGCCTGGGGAGCCCCGGGGGATTAAAGCTGGCTATGGCACCTGCAACTGCTTCCCTGAGGAGGAGAAGTGCTTCTGCGAGCCCGAAGACATCCAGGATCCACTGTGCGACGAGCTGTGCCGCACGGTGATCGCAGCCGCTGTCCTCTTCTCCTTCATCGTCTCGGTGCTGCTGTCTGCCTTCTGCATCCACTGCTACCACAAGTTTGCCCACAAGCCACCCATCTCCTCAGCTGAGATGACCTTCCGGAGGCCCGCCCAGGCCTTCCCGGTCAGCTACTCCTCTTCCGGTGCCCGCCGGCCCTCGCTGGACTCCATGGAGAACCAGGTCTCCGTGGATGCCTTCAAGATCCTGGAGGATCCAAAGTGGGAATTCCCTCGGAAGAACTTGGTTCTTGGAAAAACTCTAGGAGAAGGCGAATTTGGAAAAGTGGTCAAGGCAACGGCCTTCCATCTGAAAGGCAGAGCAGGGTACACCACGGTGGCCGTGAAGATGCTGAAAGAGAACGCCTCCCCGAGTGAGCTTCGAGACCTGCTGTCAGAGTTCAACGTCCTGAAGCAGGTCAACCACCCACATGTCATCAAATTGTATGGGGCCTGCAGCCAGGATGGCCCGCTCCTCCTCATCGTGGAGTACGCCAAATACGGCTCCCTGCGGGGCTTCCTCCGCGAGAGCCGCAAAGTGGGGCCTGGCTACCTGGGCAGTGGAGGCAGCCGCAACTCCAGCTCCCTGGACCACCCGGATGAGCGGGCCCTCACCATGGGCGACCTCATCTCATTTGCCTGGCAGATCTCACAGGGGATGCAGTATCTGGCCGAGATGAAGCTCGTTCATCGGGACTTGGCAGCCAGAAACATCCTGGTAGCTGAGGGGCGGAAGATGAAGATTTCGGATTTCGGCTTGTCCCGAGATGTTTATGAAGAGGATTCCTACGTGAAGAGGAGCCAGGGTCGGATTCCAGTTAAATGGATGGCAATTGAATCCCTTTTTGATCATATCTACACCACGCAAAGTGATGTATGGTCTTTTGGTGTCCTGCTGTGGGAGATCGTGACCCTAGGGGGAAACCCCTATCCTGGGATTCCTCCTGAGCGGCTCTTCAACCTTCTGAAGACCGGCCACCGGATGGAGAGGCCAGACAACTGCAGCGAGGAGATGTACCGCCTGATGCTGCAATGCTGGAAGCAGGAGCCGGACAAAAGGCCGGTGTTTGCGGACATCAGCAAAGACCTGGAGAAGATGATGGTTAAGAGGAGAGACTACTTGGACCTTGCGGCGTCCACTCCATCTGACTCCCTGATTTATGACGACGGCCTCTCAGAGGAGGAGACACCGCTGGTGGACTGTAATAATGCCCCCCTCCCTCGAGCCCTCCCTTCCACATGGATTGAAAACAAACTCTATGGTAGAATTTCCCATGCATTTACTAGATTCACCGGTGGAAAACTGGAAGTCGAGGGAGTGCAGGTGGAGACTATCTCCCCAGGAGACGGGCGCACCTTCCCCAAGCGCGGCCAGACCTGCGTGGTGCACTACACCGGGATGCTTGAAGATGGAAAGAAAGTTGATTCCTCCCGGGACAGAAACAAGCCCTTTAAGTTTATGCTAGGCAAGCAGGAGGTGATCCGAGGCTGGGAAGAAGGGGTTGCCCAGATGAGTGTGGGTCAGAGAGCCAAACTGACTATATCTCCAGATTATGCCTATGGTGCCACTGGGCACCCAGGCATCATCCCACCACATGCCACTCTCGTCTTCGATGTGGAGCTTCTAAAACTGGAATCTGGCGGTACCGGTGGATCCGGAGTCGACTATCCGTACGACGTACCAGACTACGCACTCGACTAA |
