# Supplementary material for: Optogenetic delivery of trophic signals in a genetic model of Parkinson’s disease
Source: PLoS Genet. 2021 Apr 15;17(4):e1009479. doi: 10.1371/journal.pgen.1009479 (PMC8049241; doi:10.1371/journal.pgen.1009479)
Supplement: S4 Table — (DOCX) [file pgen.1009479.s013.docx]

|  | **SEX CHR** | **CHR 2** | **CHR 3** |
| --- | --- | --- | --- |
| Fig 2B,H-I |  | *UAS-dRET::AU1LOV/+* |  |
| Fig 2C,H-I |  | *GMR-GAL4/+* | *UAS_dRET^MEN2B^::AU1LOV/+* |
| Fig 2D,E,H-I |  | *GMR-GAL4/+* |  |
| Fig 2F-I |  | *GMR-GAL4/UAS-dRET::AU1LOV* |  |
| Fig 3B | *PINK1^B9^/+* |  | *MEF2-GAL4/+* |
| Fig 3B,C | *PINK1^B9^/Y* |  | *MEF2-GAL4/+* |
| Fig 3B,C | *PINK1^B9^/Y* | *UAS-dRET::AU1LOV/+* | *MEF2-GAL4/+* |
| Fig 3B | *PINK1^B9^/Y* |  | *MEF2-GAL4/UAS_dRET^MEN2B^::AU1LOV* |
| Fig 3C | *+/Y* |  |  |
| Fig 4A,B,F | *PINK1^B9^/+* |  | *MEF2-GAL4/+* |
| Fig 4A,C,F | *PINK1^B9^/Y* |  | *MEF2-GAL4/+* |
| Fig 4A,D-F | *PINK1^B9^/Y* | *UAS-dRET::AU1LOV/+* | *MEF2-GAL4/+* |
| S4 Fig |  | *GMR-GAL4/UAS-dRET::AU1LOV* |  |
| S6 Fig | *PINK1^B9^/Y* |  |  |
| S7 Fig | *PINK1^B9^/Y* |  | *MEF2-GAL4/+* |
| S8 Fig | *PINK1^B9^/+* |  | *MEF2-GAL4/+* |
| S8 Fig | *PINK1^B9^/Y* |  | *MEF2-GAL4/+* |
